# Public awareness and knowledge of sepsis: a cross-sectional survey of adults in Canada

**DOI:** 10.1186/s13054-022-04215-6

**Published:** 2022-11-03

**Authors:** Jeanna Parsons Leigh, Rebecca Brundin-Mather, Stephana Julia Moss, Angie Nickel, Ariana Parolini, Deirdre Walsh, Blair L. Bigham, Alix J. E. Carter, Alison Fox-Robichaud, Kirsten M. Fiest

**Affiliations:** 1 Faculty of Health, School of Health Administration, Dalhousie University, Halifax, NS Canada; 2 Department of Critical Care Medicine, Dalhousie University, Halifax, NS Canada; 3 Department of Critical Care Medicine, University of Calgary, Calgary, AB Canada; 4 Department of Critical Care, CRISMA Center, University of Pittsburgh, Pittsburgh, PA 15260 USA; 5 Division of Critical Care, Department of Anesthesia, Stanford University, Stanford, CA USA; 6 Department of Emergency Medicine, Dalhousie University, Halifax, NS Canada; 7 Emergency Health Services Nova Scotia, Halifax, NS Canada; 8 Division of Critical Care, Department of Medicine, McMaster University, Hamilton, ON Canada; 9 Hamilton Health Sciences, Hamilton, ON Canada; 10 Hotchkiss Brain Institute, Cumming School of Medicine, University of Calgary, Calgary, Canada

**Keywords:** Sepsis, Surveys and questionnaires, Awareness, Knowledge, Critical care, Public health

## Abstract

**Background:**

Sepsis is a life-threatening complication of the body’s response to infection. The financial, medical, and psychological costs of sepsis to individuals and to the healthcare system are high. Most sepsis cases originate in the community, making public awareness of sepsis essential to early diagnosis and treatment. There has been no comprehensive examination of adult’s sepsis knowledge in Canada.

**Methods:**

We administered an online structured survey to English- or French-literate adults in Canada. The questionnaire comprised 28 questions in three domains: awareness, knowledge, and information access. Sampling was stratified by age, sex, and geography and weighted to 2016 census data. We used descriptive statistics to summarize responses; demographic differences were tested using the Rao–Scott correction for weighted chi-squared tests and associations using multiple variable regression.

**Results:**

Sixty-one percent of 3200 adults sampled had heard of sepsis. Awareness differed by respondent’s residential region, sex, education, and ethnic group (*p* < 0.001, all). The odds of having heard of sepsis were higher for females, older adults, respondents with some or completed college/university education, and respondents who self-identified as Black, White, or of mixed ethnicity (*p* < 0.01, all). Respondent’s knowledge of sepsis definitions, symptoms, risk factors, and prevention measures was generally low (53.0%, 31.5%, 16.5%, and 36.3%, respectively). Only 25% of respondents recognized vaccination as a preventive strategy. The strongest predictors of sepsis knowledge were previous exposure to sepsis, healthcare employment, female sex, and a college/university education (*p* < 0.001, all). Respondents most frequently reported hearing about sepsis through television (27.7%) and preferred to learn about sepsis from healthcare providers (53.1%).

**Conclusions:**

Sepsis can quickly cause life-altering physical and psychological effects and 39% of adults sampled in Canada have not heard of it. Critically, a minority (32%) knew about signs, risk factors, and strategies to lower risk. Education initiatives should focus messaging on infection prevention, employ broad media strategies, and use primary healthcare providers to disseminate evidence-based information. Future work could explore whether efforts to raise public awareness of sepsis might be bolstered or hindered by current discourse around COVID-19, particularly those centered on vaccination.

**Supplementary Information:**

The online version contains supplementary material available at 10.1186/s13054-022-04215-6.

## Background

Sepsis is a complication of the body’s response to an infection that can lead to life-threatening injury to organs and tissues [1]. In 2017, sepsis accounted for almost 20% of all deaths worldwide; it is a leading cause of in-hospital death in the USA and Canada [2–4]. Sepsis accounts for billions of dollars in hospitalization management costs [2, 4] and costs due to the long-term sequelae of neurocognitive, psychological, physical, and medical complications [5–7]. Estimates suggest this has increased since the emergence of severe acute respiratory syndrome coronavirus 2 (SARS-Cov-2) [8, 9]; critically ill patients with severe coronavirus disease 2019 (COVID-19) have sepsis [10, 11]. Moreover, long-term morbidities documented in many COVID-19 survivors are similar to those previously described in sepsis survivors [8].

Early recognition of the signs and symptoms of sepsis is crucial to provide rapid treatment and improve survival rates and long-term patient outcomes [12, 13]. Evidence-based clinical guidelines and practices bundles (e.g., the Surviving Sepsis International Guidelines for Management of Sepsis and Septic Shock that provides guidance caring for adult patients [14] and children [15] with sepsis or septic shock) have been central to hospital-based approaches to improve rapid sepsis diagnosis and response. However, most sepsis cases originate in the community [16], making awareness of sepsis in outpatient settings and in the public essential to improvement efforts [13, 17, 18]. Research to date suggests that sepsis is not commonly known by the general public. In 2022, we published a scoping review to identify and map the literature related to sepsis awareness, general knowledge, and information-seeking behaviors among patients, public, and healthcare professionals [19]. There was wide global variation in the proportions of the public who had heard of the term sepsis, ranging from 4 percent in France [20] to 88 percent in Germany [21]. In most public-focused studies, less than 50% of participants were self-reportedly aware of sepsis, although public awareness seemed to have gradually improved over time [19]. This may be due to increasing numbers of government (e.g., Centers for Disease Control and Prevention (CDC)) and non-government (e.g., Global Sepsis Alliance) educational campaigns [22].

What adults in Canada may know and understand about sepsis has not been comprehensively examined. The purpose of this study was to assess awareness and knowledge of sepsis as well as exposure to information about sepsis in a representative national sample. In addition, we aimed to identify social and demographic factors that may be associated with sepsis awareness and knowledge. This study is part of the collaborative work of a national multidisciplinary network of patient partners, clinical experts, and researchers (Sepsis Canada) dedicated to reducing the burden of sepsis for all people in Canada [23].

## Methods

We developed an anonymous, voluntary 10-min cross-sectional survey and contracted Leger, a Canadian-based market research and analytics company (https://leger360.com), to program, translate (to French), and administer the survey online to a representative sample of English- and French-literate adults 18 years of age or older who resided in Canada. Using an established polling firm allowed us to increase our recruitment reach and efficiency to fulfill quota sampling based on key demographic strata (age, sex, and region). All methods were performed in accordance with the guidelines and regulations of the Research Ethics Boards at Dalhousie University (#2021-0950) and the University of Calgary (#21-5708) which granted ethical approval. Prior to entering the survey, respondents reviewed an informed consent page; consent was implied by submitting the survey. The reporting of survey methods followed the Checklist for Reporting Results of Internet E-Surveys (CHERRIES) [24], available in Additional file 1.

### Questionnaire development and testing

We first created a preliminary list of questions from published articles identified in our scoping review of publications examining public awareness and knowledge of sepsis [19] and mapped them into three primary content domains: (1) awareness of sepsis, (2) sepsis information access, and (3) knowledge of sepsis. Questions were then iteratively revised by the core survey development team (three researchers (JPL, RBM, SM) and three citizen partners (AP, AN, DW)—1 sepsis patient, 1 sepsis patient family member, and 1 trained patient researcher) [25]. We subsequently invited five members of Sepsis Canada to assess the questionnaire format, content, clarity, and flow [26]. Supplementary details describing the survey development and format as well as the English version of the survey are in Additional file 2. We pilot-tested the functionality of the questionnaire with 30 respondents to ascertain the time-to-completion and logic functionality. No changes to the questionnaire were required after the pilot, and we therefore included all pilot data in the final dataset.

### Questionnaire administration

The final questionnaire was distributed in English and French via electronic mail or push notification through Leger’s proprietary Leger Opinion (LEO) panel. Leger’s LEO panel is an online pool of approximately 400,000 adults (≥ 18 years) recruited and validated through multiple methods and who consented to be contacted for research purposes. As a general population survey, all individual panel members met our inclusion criteria—18 years of age or older, lived in Canada, able to read English or French, able to provide informed consent—and had an equal chance to be randomly sampled and invited to participate. Respondents received LEO reward points after completing the questionnaire, which could be redeemed for gift cards and merchandise.

To minimize respondent straight-lining (i.e., giving the same response or a predictable pattern of responses to a series of grouped questions that use the same rating scale) to improve data quality, respondents were presented with a single question per screen and attention checks (innocuous questions with a single correct answer) were randomly inserted throughout the questionnaire. In addition, the order of the response options was randomized to reduce response selection bias. Respondents were unable to change their answers through a back button.

### Sample size calculations

We derived a minimum sample size estimate of 385 participants based on a normal approximation to the binomial distribution with a finite population correction applied [27] (assuming an observed proportion of respondents selecting a specific response option of 50%) that incorporated population size (~ 36.3 million in Canada), a 95% confidence level (95% CI) and a margin of error of 5%. We elected to collect 3,200 questionnaires to allow analyses by sociodemographic categories and calculated the associated margin of error to be ± 2.2% at a 95% CI.

### Data analysis

All quantitative data analyses were conducted using SAS 9.4 with a statistical significance set at ≤ 0.05.

We used descriptive statistics to summarize responses. Weighting is a common technique in survey analysis to statistically correct for unequal probabilities that commonly occur during sampling. In our study we used the Random Interactive Method that permitted adjusting for multiple characteristics while keeping each proportionate as a whole. The weights for each age, sex, and region categories were calculated based on current census population distributions [28]; an overall correction for each province and territory was then applied. Overall differences between sepsis awareness categories (heard of sepsis, not heard of sepsis) and between demographic categories were tested using the Rao–Scott correction to chi-squared test for weighted categorical survey data. Demographic categories include the following: (1) sex (female, male), (2) age in years (18–29, 30–44, 45–64 years, and 65 and older); (3) education (high school diploma or less, trade or vocational certification or some post-secondary college or university, post-secondary college or university); (4) annual household income (< $60,000, $60,000– < $125,000, ≥ $125,000), (5) Ethnicity (Asian East/Southeast, Asian South/Indian Caribbean, Black, Indigenous, Latin American, Middle Eastern, White, Mixed/Other Race/Ethnicity (coded as respondents who checked multiple categories across ethnic groups or checked Other), and (6) Region (British Columbia, Alberta, Saskatchewan/Manitoba, Ontario, Quebec, Atlantic Canada, Territories). Respondents who could not be classified into these categories were not included in the analyses. Finally, we calculated a continuous composite knowledge score for each of the four knowledge topic areas as well as a total sepsis knowledge score based on the summation of correctly identified response options to each knowledge question (see Additional file 3). Correctly selected response options were coded as 1; incorrect and ‘don’t know’ response options were coded as 0 (i.e., respondents were not penalized for selecting incorrect answers). We converted the knowledge sum to a percent score (0–100) for each respondent and calculated averages for the total sample.

Given the limited evidence base examining factors associated with public understanding of sepsis, backward stepwise regression was used to assess the association between sepsis awareness (dichotomous variable) and demographics, as well as to identify possible demographic or health-related predictors of total sepsis knowledge (continuous variable). The full models were fitted with all respective variables and model selection was conducted using an elimination stopping rule set at a *p* value < 0.1. ‘Prefer not to answer’ and ‘I don’t know’ response options were excluded. Demographic reference categories for both regression analyses were male (sex), 18–29 years (age), high school or less (education), < $60 K (income), and Asian East/Southeast (Ethnicity). Age was treated as a continuous variable. Health-related variables included in the sepsis knowledge full model were binary and measured the presence/absence of chronic health conditions, employment in health care, and previous sepsis experience.

## Results

### Respondents

Participation invitations were emailed to 20,791 panelists; sampling quotas were met in 3 weeks (October 14, 2021–November 4, 2021). Of the 3594 individuals who started the survey, 394 (11%) exited before completing it; the remaining 3200 (89%) completed all questions and were included in the analysis. We received 79% of surveys in English and 21% in French. The sample was approximately equal females (48.1%) and males (51.3%), with a weighted mean age of 48 years (Standard Deviation = 17.2). A summary of respondent characteristics is presented in Table 1.

**Table 1:** Respondent characteristics (*n* = 3200)

Characteristic	Unweighted number (%)^a^	Weighted %
Gender	3189 (99.7)	
Woman	1588 (49.8)	51.3
Man	1581 (49.6)	48.1
Self-described^b^	20 (0.6)	0.6
Sex	3,191 (99.7)	
Female	1598 (50.1)	51.5
Male	1593 (49.9)	48.5
Age	3200 (100)	
Mean (SD)	48.4 (16.6)	48.2 (17.2)
18–29	573 (17.9)	17.9
30–44	817 (25.5)	25.5
45–64	1133 (35.4)	35.4
65 or older	677 (21.2)	21.2
Region	3200 (100)	
British Columbia	303 (9.5)	13.5
Alberta	263 (8.2)	11.2
Manitoba/Saskatchewan	242 (7.6)	6.5
Ontario	1219 (38.1)	38.3
Québec	761 (23.8)	23.4
Atlantic^c^	212 (6.4)	6.8
Territories^d^	200 (6.2)	0.3
City size	3165 (98.9)	
Small town or city (up to 10,000 people)	497 (15.7)	14.7
Medium size city (> 10,000 to < 100,000)	839 (26.5)	24.3
Large city (> 100,000–1,000,000)	1058 (33.4)	34.6
Large metropolitan area (> 1,000,000)	771 (24.4)	25.9
Ethnicity/race^e^	3,119 (97.5)	
Single response	2,828 (90.7)	
Multiple response	291 (9.3)	
Black, indigenous, person of color	670 (21.5)	
Asian East/Southeast	237 (7.6)	8.6
Asian South/Indian Caribbean	92 (2.9)	3.2
Black	89 (2.9)	3.0
Indigenous	71 (2.3)	1.7
Latin American	27 (0.9)	0.9
Middle eastern	26 (0.8)	0.9
Mixed/other	128 (4.1)	4.0
White	2449 (78.5)	77.8
Highest education	3173 (99.2)	
High school or less	529 (16.5)	18.0
CEGEP, trade, or technical college	507 (15.8)	15.1
Some college/university	409 (12.8)	16.7
College/university degree (s)	1728 (54.0)	41.7
Household income	2854 (89.2)	
0$–$59,999	975 (34.2)	36.0
$60,000–$124,999	1231 (43.1)	41.8
$125,000 or more	648 (22.7)	20.7
Employment		
Employment status^f^	3166 (98.9)	
Employed (full-time/part-time hours)	1911 (60.4)	57.4
Not employed^g^	501 (15.8)	5.2
Retired	783 (24.7)	25.8
Other	56 (1.8)	1.7
Employment sector	3131 (97.8)	–
Animal careers	22 (0.7)	1.4
Aviation	31 (1.0)	1.6
Arts	28 (0.9)	1.6
Business	194 (6.2)	10.9
Education	213 (6.8)	11.0
Law enforcement	38 (1.2)	1.9
Media	26 (0.8)	1.7
Medical/health	235 (7.5)	12.5
Service industry	294 (9.4)	1.1
STEM careers	201 (6.4)	16.3
Other	530 (16.9)	10.9
Diagnosis with chronic health condition (s)^g^	3107 (97.1)	
Yes, current diagnosis	1189 (38.3)	40.0
No, current diagnosis	1918 (61.7)	60.0

Abbreviations: *CEGEP* Collège d'enseignement général et professionnel (a publicly funded college providing technical, academic, vocational or a mix of programs in the province of Quebec); *NWT* northwest territories, *STEM* Science, technology, engineering, and math

^a^Frequencies and percent are noted unless otherwise indicated. Prefer not to answer response options are excluded from percent analyses and individual N reported

^b^Self-described includes non-binary, two-spirited, and prefer to self-describe

^c^Atlantic Canada includes the provinces of New Brunswick, Newfoundland and Labrador, Nova Scotia, and Prince Edward Island

^d^Territories include the territories of Yukon, Northwest Territories, and Nunavut

^e^The following categories were combined: (1) Asian East (e.g., Chinese, Japanese, Korean) and Asian Southeast (e.g., Malaysian, Filipino, Vietnamese); (2) Asian South (e.g., Indian, Pakistani, Sri Lankan) and Indian Caribbean; (3) Black-African (e.g., Ghanian, Kenyan, Somali), Black-Caribbean (E.g., Barbadian, Jamaican), Black-North American (e.g., Canadian, American) into Black; (4) Indigenous, Inuit, First Nations, and Métis into ‘Indigenous’; and (5) White-European (e.g., English, Italian, Portuguese Russian), White-North American (e.g., Canadian, American) into White. Respondents who checked multiple categories across recoded groups were coded as Mixed; mixed was combined with open-ended ‘Other’ responses

^f^Includes student, full-time parent or homemaker, unemployed or unable to work for any reason

^g^Participants checked if diagnosed any condition on a predefined list that included autoimmune disease, cancer, diabetes, cardiovascular disease, obesity, other chronic diseases (e.g., high cholesterol, kidney disease), and Other

### Awareness of sepsis

**Fig. 1:**
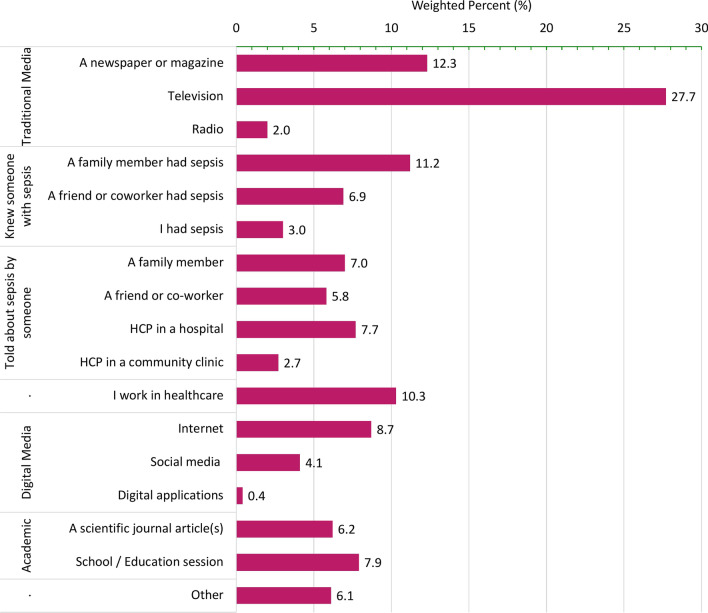
Sources of sepsis awareness. Response options applied to me survey question ''How did you hear about sepsis?" which was asked only to respondents who answered, 'Yes' to the question 'Have you ever heard of the medical condition sepsis?' (n=l978). Respondents who answered, "I don't remember sepsis" (n=352) were excluded from the denominator. The response option, Digital applications, was truncated from the application downloaded on my cellphone or tablet.' HCP, Healthcare Provider

Additional file 4: Table S1 presents the results of all awareness items on our survey. Overall, 61.4% of our sample reported that they had heard of the medical condition called sepsis; 32% had not heard of sepsis, and 6% were uncertain. There was significant regional variation in self-reported awareness (*p* < 0.001) with respondents from Quebec least frequently (46%) and respondents from the Territories most frequently (73.1%) having heard of sepsis (Additional file 4: Figure S1). One-fifth (21.6%) of our sample reported that they knew someone with sepsis, the highest proportions identifying a relative other than immediate family (21.9%) or a friend (21.1%) (Additional file 4: Table S1). Respondents who had heard of sepsis most frequently reported hearing about it from traditional media sources (41.9%) (Additional file 4: Table S1), particularly television (27.7%) (Fig. 1).

**Fig. 2:**
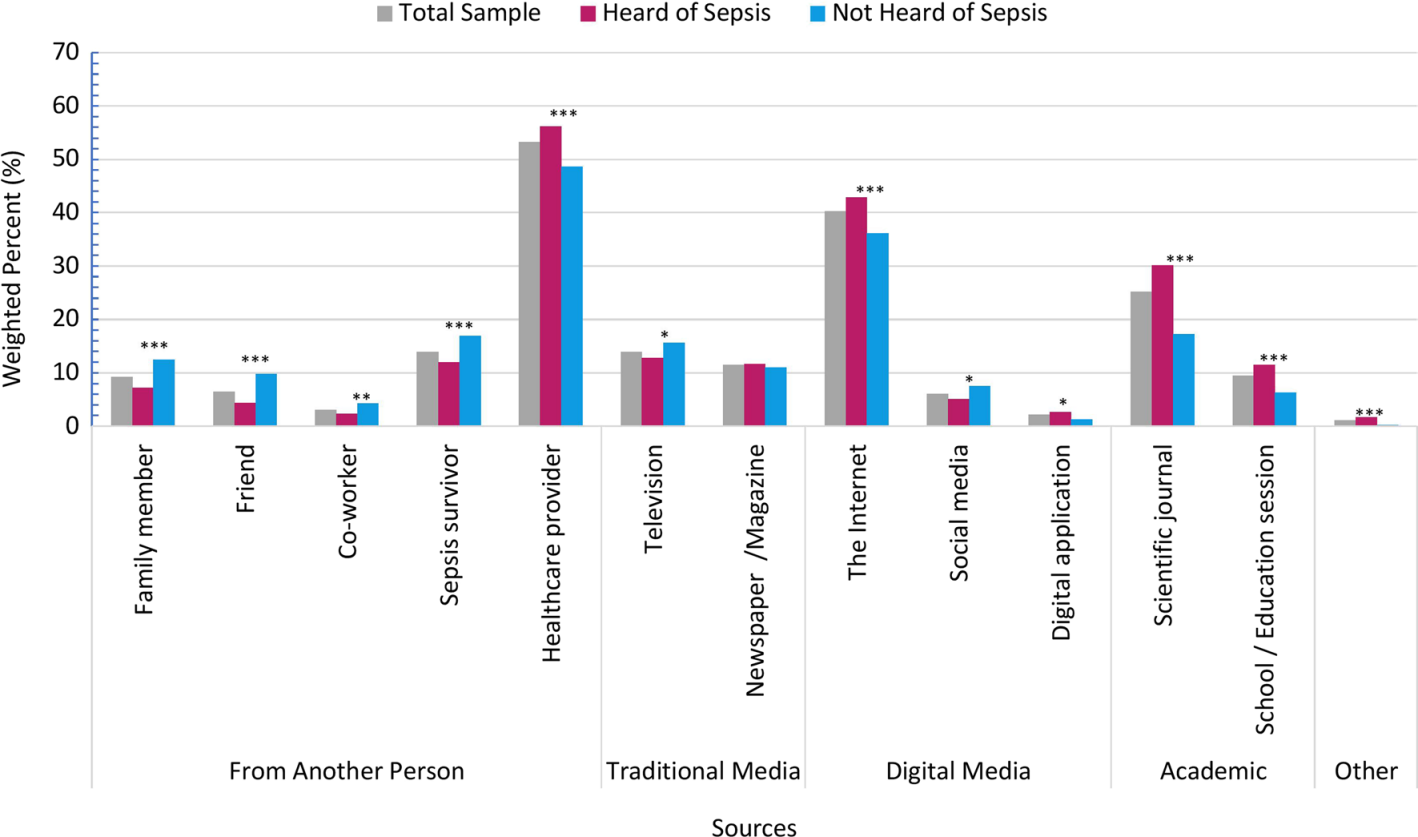
Preferred sources to learn about sepsis. Response options applied to me survey question ''How did you hear about sepsis?" which was asked only to respondents who answered, 'Yes' to the question 'Have you ever heard of the medical condition sepsis?" (n=l978). Respondents who answered, "I don't remember how I heard about sepsis" (n=3S2) were excluded from the denominator. The response option, Digital applications, was truncated from the response option 'An application downloaded on my cellphone or tablet.' HCP. Healthcare Provider

Only a small minority of respondents overall (5.7%) indicated that they would not like to learn about sepsis. Among those who identified how they would like to learn about sepsis (*n* = 85.0%), most selected a healthcare provider (53.3%), followed by the Internet (40.1%) and scientific journals (25.2%) (Fig. 2). The top preferred sources were the same regardless of whether respondents had previously heard of sepsis or not (Fig. 2), although there were significant proportional differences between the two groups (Fig. 2).

Sepsis awareness was significantly associated with respondents’ education, ethnicity, sex, and age (Table 2). Respondents with some college or university education and those with a degree had greater odds of having heard of sepsis (Odds Ratio (OR) 2.25, 95% CI 1.636, 3.087, and OR = 2.80, 95% CI 2.21, 3.56, respectively) compared to respondents with high school education. Respondents who identified as mixed ethnicity had nearly 5 times the odds (OR = 4.75, 95% CI 2.77, 8.67) of having heard of sepsis than respondents self-identifying as East/Southeast Asian, while those who identified as white (OR = 3.164, 95% CI 2.247, 4.455) or black (OR = 2.161, 95% CI 1.235, 3.782) had, respectively, three and two times the odds of having heard of sepsis compared to East/Southeast Asian respondents. Males had half the odds of having heard of sepsis than females (OR = 0.51, 95% CI 0.43, 0.60).

**Table 2:** Odds of having heard of the medical condition sepsis given demographic characteristics

Covariate	Categories	Overall *p* value	OR	95% CI	*p* value
Age		0.005	1.008	1.002, 1.013	0.005
Education	High school or less (Reference Category)	0.000			
	CEGEP/Vocational college/Trade		1.224	0.916, 1.637	0.171
	Some College or University (no degree)		2.249	1.637, 3.089	0.000
	College/University degree(s)		2.798	2.209, 3.545	0.000
Ethnicity	Asian East/Southeast (Reference Category)	0.000			
	Asian South/Indian Caribbean		1.646	0.917, 2.955	0.095
	Black		2.161	1.235, 3.782	0.007
	Latin American		2.337	0.925, 5.907	0.073
	Middle Eastern		0.667	0.215, 2.065	0.482
	Mixed/Other		4.751	2.700, 8.357	0.000
	White		3.164	2.247, 4.455	0.000
Sex	Female (Reference Category)	0.000			
	Male		0.508	0.428, 0.604	0.000

Logistic regression model selection was conducted from the fitted model using backward stepwise selection with elimination stopping rule set to *p* value < 0.1. Response options ‘Prefer not to answer’ and ‘I don’t know’ from each independent variable were excluded from the dataset resulting in a sample size of 2799.The overall *p* value was obtained from each logistic regression analysis and may be interpreted at the statistical significance of the model given all covariates in the model are held constant

*CEGEP* Collège d'enseignement général et professionnel (a publicly funded college providing technical, academic, vocational or a mix of programs in the province of Quebec), *CI* confidence interval, *OR* odds ratio

### Knowledge of sepsis

**Fig. 3:**
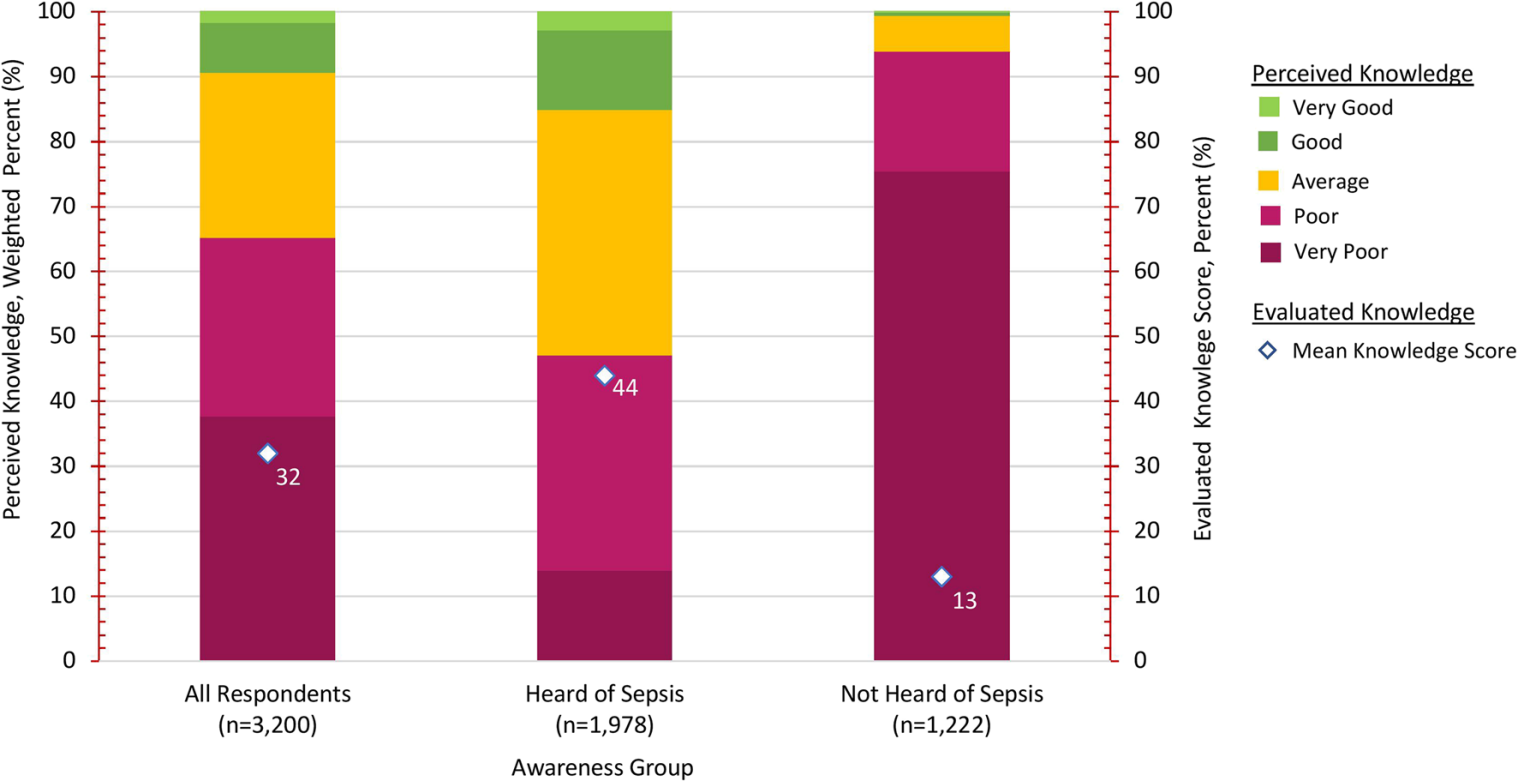
Level of perceived knowledge and evaluated knowledge by sepsis awareness category. Perceived level of knowledge was defined by a 5-point Likert scale question "How would you rate your level of knowledge about sepsis?" (l = very poor, 2 = poor, 3 = average, 4 = good, 5 = very good). Mean 'Total Knowledge' score was calculated by adding each respondents correct identification of response options to each knowledge question. Correctly selected responses options were coded as 1; incorrect and "don't know" response options were coded as 0. The total number of possible correct answers was 26. The mean score for each respondent was converted to a percent ranging from 0 to 100. Sepsis Awareness category g was coded to the question "Have you ever heard of the medical condition sepsis?" with responses of 'Yes' coded as 'Heard of Sepsis' (n = l978), and 'No' or 'Uncertain' coded as 'Not Heard of Sepsis' (n = l222).

Relatively few (9.5%) respondents overall perceived their level of knowledge about sepsis as good (7.7%) or very good (1.8%) (Fig. 3), though higher proportions of respondents who had heard of sepsis rated their knowledge as good or very good (15.1%) than respondents who had not heard of sepsis (0.5%) (*p* < 0.001) (Fig. 3). Similarly, respondents who had heard of sepsis were able to identify on average 44% of all correct responses (*n* = 26) on knowledge-based questions (mean = 44.3%, SD = 18.9%) compared to respondents who had not heard of sepsis who identified an average of 13% of responses (mean = 12.9%, SD = 18.4%) (Fig. 3). Perceptions and knowledge also varied across demographic groups (see Additional file 5: Figure S1).

**Fig. 4:**
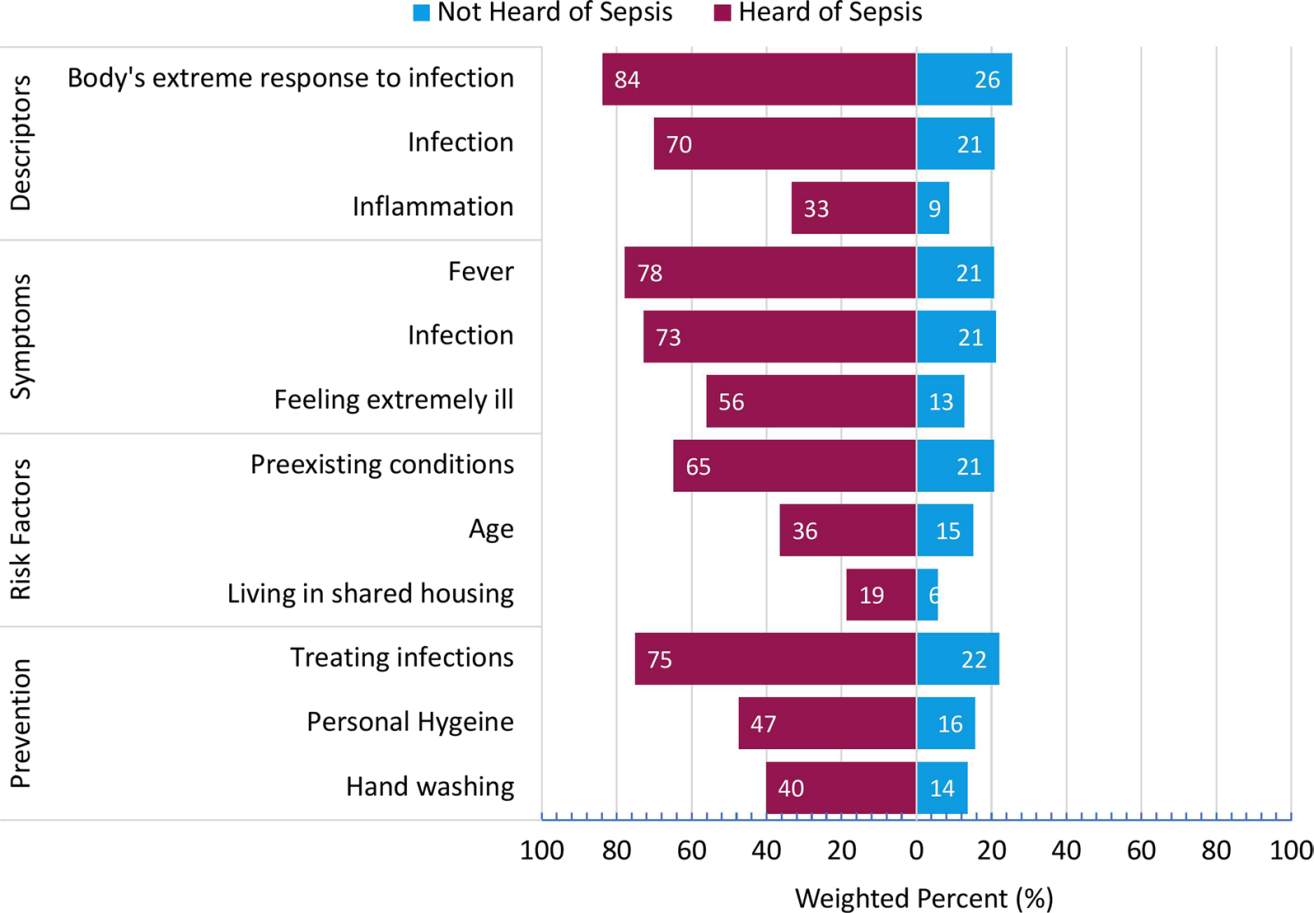
Top three correct responses selected within each knowledge area. Sepsis Awareness category was coded to the question "Have you ever heard of the medical condition sepsis?” with responses of 'Yes' coded as 'Heard of Sepsis' (n = 1978), and 'No' or 'Uncertain' coded as 'Not Heard of Sepsis' (n=1222). Descriptors were identified from the multiple-response question "Select the word (s) or phrase (s) that describe sepsis? Symptoms were selected from the multiple-response question, "Which of the following, if any, are common symptoms or signs of sepsis?" Risk factors were selected from the multiple-response question, "Which of the following factors are associated with a higher risk of a person developing sepsis?"; Prevention items were selected from the multiple-response question, "Which of the following actions, if any, can help prevent or lower your risk of developing sepsis?" Chi-square tests showed significance differences between awareness categories 'Heard of Sepsis' and 'Not Heard of Sepsis' at D <.001 for all knowledge items in Table 2.

Additional file 5: Table S1 presents the distribution of responses to all knowledge items (*n* = 26). Overall, one-fifth (21.3%) of our sample were not able to identify a correct answer to any knowledge question. Few respondents overall selected clearly false answers. About one-third of all knowledge items were answered correctly (Mean = 32.3%, SD = 24.2%) though mean scores varied across the four topics including definition (Mean = 53.0%, SD = 36.3%), symptoms (Mean = 31.5%, SD = 28.5%), risk factors (Mean = 16.5%, SD = 18.3%), and prevention (Mean = 36.3%, SD = 37.1%) (Additional file 5: Table S1). Respondents who had heard of sepsis generally identified correct responses more frequently (*p* < .001) than respondents who had not heard of sepsis (Fig. 4). Overall, the top recognized descriptor of sepsis was ‘the body’s extreme response to infection’ (61.3%) and top recognized sign/symptom of sepsis was ‘fever’ (55.7%) (Additional file 5: Table S1). Key signs of sepsis, such as ‘fast breathing (21.2%) and passing no urine all day (8.0%) were less frequently identified, as was an understanding that conditions such as COVID-19 (21.3%) and influenza (16.4%) contribute to a higher risk of developing sepsis (Additional file 5: Table S1).

Respondents understanding of sepsis prevention measures was generally poor (Mean = 36.6%; SD = 37.1%), although 60% of respondents correctly identified at least one of four listed actions that could ‘*help prevent or lower (their) risk of developing sepsis*.’ The most frequently selected action was treating infections (54.6%), followed by personal hygiene (35.1%), and hand washing (30.0%) (Additional file 5; Table S1). Only one-quarter of respondents (25.1%) identified keeping vaccinations up to date as a prevention strategy. Respondents who did not initially select vaccination as a prevention measure were subsequently asked if it was true or false that vaccination for (1) seasonal influenza (i.e., the flu), and (2) SARS-CoV-2 could decrease their risk of developing sepsis; only 7.5% and 6.9% of respondents, respectively, selected true. Most respondents indicated that they would seek medical support from an urgent care or hospital emergency department (78.4%) or phone the emergency line (30.9%) if someone close to them became ill with symptoms of sepsis (Additional file 5; Table S1).

### Predictors of sepsis knowledge

Of the eight variables, we hypothesized might be associated with sepsis knowledge (possible score range from 0 to 26), the strongest predictors were having prior experience of sepsis (*β* = 7.711, *p* < 0.001), working in health care (*β* = 2.756, *p* = 0.001), having a college/university degree (*β* = 1.234, *p* < 0.001), and being female (*β* = − 1.092, *p* < 0.001) (Table 3). Age, chronic health conditions, and ethnicity were not significant in the full model and therefore eliminated in the backward regression.

**Table 3:** Sociodemographic predictors of sepsis knowledge

Covariate	Categories	Overall *p* value	β estimated parameter	*p* value
Education	High school or less	0.000		
	CEGEP/Vocational college/Trade		− 0.028	0.932
	Some College/University (no degree)		0.803	0.033
	College/University degree (s)		1.234	0.000
Healthcare employment	No	0.000		
	Yes		2.756	0.000
Income	< $60 k	0.097		
	$60 k − < $125 K		− 0.226	0.325
	> = $125 K		0.328	0.245
Prior sepsis experience	No	0.000		
	Yes		7.711	0.000
Sex	Female	0.000		
	Male		− 1.092	0.000

Multiple linear regression model selection was conducted from the fitted model using backward stepwise selection with elimination stopping rule set to *p* value < 0.1. Response options ‘Prefer not to answer’ and ‘I don’t know’ from each independent variable were excluded from the dataset, resulting in a sample size of 2709. ‘Healthcare employment’ was coded from respondent’s identification of the ‘*main employment sector worked in*’ with ‘Medical/Health’ coded as ‘Yes’ and all other response options coded as ‘No’ (Animal Careers, Aviation, Arts, Business, Education, Law Enforcement, Media, Military Careers, Service Industry, Science/Technology/Engineering/ Math (STEM) Careers, Other). ‘Prior sepsis experience’ was coded as ‘Yes’ if respondents answered, ‘Yes’ to ‘*Have you heard of the medical condition sepsis*?’ or did not select ‘No, I do not personally know anyone who has had sepsis’ to the question ‘*Do you know anyone who has ever had sepsis*?’ The beta coefficient is the average difference in sepsis knowledge between the predictor group in question and the reference group, while holding other predictors in the model constant. For example, the sepsis knowledge score of those who have prior sepsis experience is expected to be on average 7.71 point higher than individuals without previous sepsis knowledge

### Accessing information on sepsis

About one-fifth (19.7%) of respondents who had heard of sepsis (61.8%) had actively looked for information about sepsis (Additional file 6); the majority (68.5%) used the Internet. Ease of information (37.1%) was the most frequently selected reason that influenced where they had actively looked for information, followed by the reliability of information (27.5%) and trust in information (22.1%). Few respondents (10%) who had actively looked for the information reported experiencing difficulties getting the information that they wanted or needed. Lack of information on the topic being searched (57.2%) and lack of explanation in plain language (45.3%) were the common difficulties cited by these respondents.

## Discussion

In this survey of adults in Canada, we found moderate self-reported awareness of sepsis overall but limited knowledge when evaluated on factual questions about sepsis. Females, older adults, and adults with college/university education were more likely to be aware of sepsis as were adults who self-identified as mixed ethnicity (i.e., self-identified across multiple ethnic categories), Black, or White. Previous experience with sepsis and working in health care were significant predictors of total sepsis knowledge, as were respondents’ sex and educational attainment. The overall lack of extensive knowledge may be related to the respondent’s primary source of awareness being television. At the same time, most respondents indicated that they wanted to learn about sepsis and preferred to learn from a credible source like a healthcare provider. This finding underscores the significant role of health professionals in fostering literacy and supports a multifaceted approach to address identified knowledge gaps [29]. In addition, given the propensity to use the Internet as a primary source of health information in general, and sepsis information specifically, web analytics tracking should be used to provide insight into information-seeking behaviors [22, 30]. Improving awareness and knowledge requires increased availability and access to quality data across all sectors.

Our study suggests greater public awareness of sepsis in Canada than in several other countries, though global survey findings have been mixed [19]. The percentages of participants who had heard of the term ‘sepsis’ in Rubulotta et al.’s early 2009 multi-country study ranged from 4% in France to 53% in Germany [20]. More recent population-based studies have also ranged in the percentage of adults aware of sepsis—e.g., 29% of sampled Irish [17], 57% of sampled Saudi [31], and 88% of sampled Germans [21]—but generally suggest a trend to greater awareness than previous decades. However, awareness among adults in our sample did not correlate with depth of knowledge. This was particularly evident for knowledge of risk factors and mortality, which most of our sample could not identify, performing lower than participants in other studies with similar data [20]. Positively, 65% of our study participants correctly recognized that sepsis was the body’s extreme response to an infection, which was higher than Rubulotta [20] and Park [32] who independently found about one-third of their participants were unable to define sepsis, despite having heard of the term.

Few participants in our study identified approaches to lower the risk of sepsis (e.g., by keeping vaccinations up to date (25%)) or identified important warning signs of sepsis (e.g., fast breathing (21%)). These knowledge areas are critical to reducing the incidence and optimizing early detection and treatment. However, detecting sepsis can be difficult as the signs are often subtle, nonspecific, and variable [13], challenging efforts to establish clear and concise expectations of what the public should know about sepsis and associated calls to action. Moreover, the public’s incomplete understanding of the role of infection control measures, like hand washing and vaccination, in reducing the severity of disease (and not just disease prevention) found in our study should be considered. Our results align with current evidence reflecting gaps in knowledge, beliefs, and attitudes among the public about vaccination, [33] a topic that has gained extraordinary public interest during the COVID-19 pandemic. COVID-19, at the forefront of public discourse on many issues, presents a unique opportunity to bring greater attention to sepsis. Many in the sepsis community are aware of the role of vaccination and possible health outcomes associated with severe or critical COVID-19 [8, 9, 34]; however, we found a general lack of knowledge on this information among the general public in Canada. The COVID-19 pandemic has highlighted the significance of (1) healthcare providers as a primary source for credible and trusted information, and (2) television and digital media as primary mediums to deliver information on health and illness [33, 35]. Addressing potential barriers to accessing healthcare professionals for knowledge acquisition on sepsis is an important next step in our work and one that we are currently investigating by conducting a series of focus groups with healthcare providers across Canada to explore how best to prompt their engagement in the education of sepsis. Reconciling how preferred and actual sources of information may intersect and be leveraged to improve public health literacy will also be vital to the success of any sepsis awareness initiative.

Health promotion initiatives aim to engage and empower individuals and communities to make informed decisions that reduce the risk of developing diseases. Campaigns to raise awareness and knowledge of specific diseases, such as the face–arm–speech–time (FAST) test for stroke [36, 37] were premised on the notion that providing the public with information will improve prevention, recognition, and prompt treatment. However, the successes of public campaigns are often equivocal. For example, extensive evaluations of stroke campaign efforts have shown increased public awareness of stroke but limited or short-lived impact on recognition and response [36–40]. Although intercountry differences in sepsis awareness have been attributed to high-profile, nationally targeted campaigns [17, 41] such as the UK Sepsis Trust campaign ‘Just Ask, Could it be Sepsis?’ [42] and the CDC campaign ‘Get Ahead of Sepsis?’ [43], few countries have conducted multiple studies over time, making it difficult to attribute shifts in public awareness to campaign impacts. The leading sepsis organization in the USA, Sepsis Alliance, has collected longitudinal data from Americans that showed improved awareness between 2003 (19%) and 2021 (65%); an increase that may be correlated with concerted public campaign efforts by the organization, but cannot be causally determined [40, 44, 45]. Our survey provides national baseline data which can be used by sepsis networks in Canada to inform knowledge translation strategies; repeat surveys will further contribute to establishing relevant, evidence-informed programs and policies. Additionally, tracking disparities in sepsis awareness and knowledge across sociodemographic factors could further inform targeted campaigns. Similar to other research our data showed that higher education attainment was associated with higher sepsis knowledge [21, 31], younger age associated with lower sepsis awareness [17], and females associated with higher sepsis awareness and knowledge. The latter finding may reflect a greater tendency of women to engage with health-related information. [46–48]

### Limitations

First, we used a cross-sectional survey design that permitted us to collect data from a large and representative sample of the Canadian population. However, as data are collected at a single point in time, results have limited longitudinal applicability and may reflect context outside our study. For example, we disseminated our survey one month after the widespread global promotion of World Sepsis Day. Mid-October was also around the time that former US president Bill Clinton was hospitalized for a urologic infection and suspected sepsis. Second, we relied on a volunteer panel of potential participants (Leger’s LEO panel) which might have introduced recruitment bias. Third, the limited knowledge in this area prohibited a priori selection of potentially relevant covariates for our regression analyses. Finally, our survey was administered exclusively online and available in Canada’s two official languages, potentially excluding otherwise eligible adults in Canada who do not access the Internet (9%) or who neither speak English nor French (1.8%) [28].)

## Conclusions

This study is the first to examine public awareness, general knowledge, and information access of sepsis in Canada. We found an incomplete awareness and understanding of sepsis among adults. Our study provides valuable benchmark data and identification of major gaps in the public’s understanding as well as contributes to global literature evaluating public engagement in sepsis information. Future work should focus on elucidating gaps in the public’s knowledge, establishing target groups and modalities for knowledge translation, and creating key messages to integrate with a national sepsis awareness program.

## Supplementary Information

**Additional file 1**. Checklist for Reporting Results of Internet E-Surveys (CHERRIES)

**Additional file 2**. Sepsis Survey Development and Format

**Additional file 3**. Sepsis Knowledge Question Scoring Key

**Additional file 4**. Awareness of Sepsis Additional Results. **Table S1**. Distribution of responses for items related to awareness of sepsis. **Figure S1**. Regional differences in awareness of sepsis and interest in learning about sepsis

**Additional file 5**. Knowledge of Sepsis Additional Results. **Figure S1**. Sepsis awareness, perceived knowledge, and evaluated knowledge by respondent characteristics. **Table S1**. Distribution of responses for items related to knowledge of sepsis.** Figure S2**. Distribution of responses seeking medical support when ill with symptoms of sepsis

**Additional file 6**. Distribution of responses for items related to sepsis information access

## Data Availability

The datasets generated and/or analyzed during the current study are not publicly available as we did not secure direct permission from the survey respondents to share the de-identified dataset with the general public. Requests for the de-identified data can be directed to the institutional research ethics boards overseeing the conduct of the study via the principal investigator, Dr. Jeanna Parsons Leigh (jparsonsleigh@dal.ca).

## Abbreviations

*β*: Beta
CDC: Centers for Disease Control and Prevention
CEGEP: Collège d'enseignement général et professionnel (a publicly funded college providing technical, academic, vocational or a mix of programs in the province of Quebec)
CI: Confidence interval
COVID-19: Coronavirus disease 2019
FAST: Face–arm–speech–time
*K*: Thousand
OR: Odds ratios
SARS-CoV-2: Severe acute respiratory syndrome coronavirus 2
SD: Standard deviation
STEM: Science, Technology, Engineering, and Math
TV: Television
WHO: World Health Organization
