# Supplementary material for: Public awareness and knowledge of sepsis: a cross-sectional survey of adults in Canada
Source: Crit Care. 2022 Nov 3;26:337. doi: 10.1186/s13054-022-04215-6 (PMC9632573; doi:10.1186/s13054-022-04215-6)
Supplement: Supplementary file 1 — Additional file 1. Checklist for Reporting Results of Internet E-Surveys (CHERRIES) [file 13054_2022_4215_MOESM1_ESM.docx]

**Additional File 1. Checklist for Reporting Results of Internet E-Surveys (CHERRIES)**

| ***Checklist Item*** | ***Explanation*** | ***Page Number*** |
| --- | --- | --- |
| Describe survey design | Describe target population, sample frame. Is the sample a convenience sample? (In “open” surveys this is most likely.)  *We developed an anonymous, voluntary 10-minute cross-sectional survey* …*administer the survey online to a representative sample of English and French literate adults 18 years of age or older who resided in Canada* | Pages 2 |
| IRB approval | Mention whether the study has been approved by an IRB.  *All methods were performed in accordance with the guidelines and regulations of the Research Ethics Boards at Dalhousie University (#2020-5121) and the University of Calgary (#20-0538) who granted ethical approval for this study.* | Page 2 |
| Informed consent | Describe the informed consent process. Where were the participants told the length of time of the survey, which data were stored and where and for how long, who the investigator was, and the purpose of the study?  *Prior to entering the survey, respondents reviewed an informed consent page; consent was implied by completing and submitting the survey.* | Page 2 |
| Data protection | If any personal information was collected or stored, describe what mechanisms were used to protect unauthorized access.  *---an anonymous, voluntary 10-minute cross-sectional survey…* | Page 2 |
| Development and testing | State how the survey was developed, including whether the usability and technical functionality of the electronic questionnaire had been tested before fielding the questionnaire.  *We first created a preliminary list of questions from published articles identified in our scoping review of publications examining public awareness and knowledge of sepsis^18^ and mapped them into three primary content domains: (1) awareness of sepsis, (2) sepsis information access, and (3) knowledge of sepsis. Questions were then iteratively revised by the core survey development team (three researchers (JPL, RBM, SM) and three citizen partners (AP, AN, DW)—1 sepsis patient, 1 sepsis patient family member, and 1 trained patient researcher).^24^ We subsequently invited five members of Sepsis Canada to assess the questionnaire format, content, clarity, and flow.^25^ Supplementary details describing the survey development and format* as well as the English version of the survey *are in Additional File 2. We pilot tested the functionality of the questionnaire with 30 respondents to ascertain the time-to-completion and logic functionality. No changes to the questionnaire were required after the pilot and we therefore included all pilot data in the final dataset.* | Page 2 |
| Open survey versus closed survey | An “open survey” is a survey open for each visitor of a site, while a closed survey is only open to a sample which the investigator knows (password-protected survey). | N/A |
| Contact mode | Indicate whether or not the initial contact with the potential participants was made on the Internet. (Investigators may also send out questionnaires by mail and allow for Web-based data entry.)  *The final questionnaire was distributed in English and French via electronic mail or push notification through Leger’s proprietary Leger Opinion (LEO) panel. Leger’s LEO panel is an online pool of approximately 400,000 adults (≥18 years) recruited and validated through multiple methods and who consented to be contacted for research purposes.* | Page 7 |
| Advertising the survey | How/where was the survey announced or advertised? Some examples are offline media (newspapers), or online (mailing lists – If yes, which ones?) or banner ads (Where were these banner ads posted and what did they look like?). It is important to know the wording of the announcement as it will heavily influence who chooses to participate. Ideally the survey announcement should be published as an appendix.  *The final questionnaire was distributed in English and French via electronic mail or push notification through Leger’s proprietary Leger Opinion (LEO) panel.* | Page 2 |
| Web/E-mail | State the type of e-survey (eg, one posted on a Web site, or one sent out through e-mail). If it is an e-mail survey, were the responses entered manually into a database, or was there an automatic method for capturing responses?  *The final questionnaire was distributed in English and French via electronic mail or push notification through Leger’s proprietary Leger Opinion (LEO) panel.* | Page 2 |
| Context | Describe the Web site (for mailing list/newsgroup) in which the survey was posted. What is the Web site about, who is visiting it, what are visitors normally looking for? Discuss to what degree the content of the Web site could pre-select the sample or influence the results. For example, a survey about vaccination on a anti-immunization Web site will have different results from a Web survey conducted on a government Web site | NA |
| Mandatory/voluntary | Was it a mandatory survey to be filled in by every visitor who wanted to enter the Web site, or was it a voluntary survey?  *We developed an anonymous, voluntary 10-minute cross-sectional survey….* | Page 2 |
| Incentives | Were any incentives offered (eg, monetary, prizes, or non-monetary incentives such as an offer to provide the survey results)?  *Respondents received LEO reward points after completing the questionnaire, which could be redeemed for gift cards and merchandise.* | Page 3 |
| Time/Date | In what timeframe were the data collected?  *Participation invitations were emailed to 20,791 panelists; sampling quotas were met in 3 weeks (Oct 14, 2021-Nov 4th, 2021).* | Page 3 |
| Randomization of items or questionnaires | To prevent biases items can be randomized or alternated.  *In addition, the order of the response options was randomized to reduce response selection bias. Respondents were unable to change their answers through a back button.* | Page 3 |
| Adaptive questioning | Use adaptive questioning (certain items, or only conditionally displayed based on responses to other items) to reduce number and complexity of the questions.  *We pilot tested the functionality of the questionnaire with 30 respondents to ascertain the time-to-completion and logic functionality.* | Page 3 |
| Number of Items | What was the number of questionnaire items per page? The number of items is an important factor for the completion rate.  *To minimize respondent straight lining (i.e., giving the same response or a predictable pattern of responses to a series of grouped questions that use the same rating scale) to improve data quality, respondents were presented with a single question per screen and attention checks (innocuous questions with a single correct answer) were randomly inserted throughout the questionnaire* | Page 3 |
| Number of screens (pages) | Over how many pages was the questionnaire distributed? The number of items is an important factor for the completion rate.  *The final questionnaire comprised of 28 questions measuring the three content domains—sepsis awareness (n=3), sepsis information and access (n=7), and sepsis knowledge (n=18)—and a 17-item demographics section (e.g., age, gender, ethnicity, education).* | Additional File 2 |
| Completeness check | It is technically possible to do consistency or completeness checks before the questionnaire is submitted. Was this done, and if “yes”, how (usually JAVAScript)? An alternative is to check for completeness after the questionnaire has been submitted (and highlight mandatory items). If this has been done, it should be reported. All items should provide a non-response option such as “not applicable” or “rather not say”, and selection of one response option should be enforced.  *Of the 3,594 individuals who started the survey, 394 (10.96%) exited before completing it; the remaining 3,200 (89.03%) completed all questions and were included in the analysis.* | Page 3 |
| Review step | State whether respondents were able to review and change their answers (eg, through a Back button or a Review step which displays a summary of the responses and asks the respondents if they are correct).  *Respondents were unable to change their answers through a back button.;* | Page 3 |
| Unique site visitor | If you provide view rates or participation rates, you need to define how you determined a unique visitor. There are different techniques available, based on IP addresses or cookies or both.  *Duplicate entries were avoided by preventing access to the survey if the LEO panelist was already registered in the LEO database as having completed the survey.* | Additonal File 2 |
| View rate (Ratio of unique survey visitors/unique site visitors) | Requires counting unique visitors to the first page of the survey, divided by the number of unique site visitors (not page views!). It is not unusual to have view rates of less than 0.1 % if the survey is voluntary. | Not calculated |
| Participation rate (Ratio of unique visitors who agreed to participate/unique first survey page visitors) | Count the unique number of people who filled in the first survey page (or agreed to participate, for example by checking a checkbox), divided by visitors who visit the first page of the survey (or the informed consents page, if present). This can also be called “recruitment” rate.  *Participation invitations were emailed to 20,791 panelists; 3,594 individuals started the survey* (17.29%) | Page 3 |
| Completion rate (Ratio of users who finished the survey/users who agreed to participate) | The number of people submitting the last questionnaire page, divided by the number of people who agreed to participate (or submitted the first survey page). This is only relevant if there is a separate “informed consent” page or if the survey goes over several pages. This is a measure for attrition. Note that “completion” can involve leaving questionnaire items blank. This is not a measure for how completely questionnaires were filled in. (If you need a measure for this, use the word “completeness rate”.)  *Of the 3,594 individuals who started the survey, 394 (10.96%) exited before completing it; the remaining 3,200 (89.03%) completed all questions and were included in the analysis.* | Page3 |
| Cookies used | Indicate whether cookies were used to assign a unique user identifier to each client computer. If so, mention the page on which the cookie was set and read, and how long the cookie was valid. Were duplicate entries avoided by preventing users access to the survey twice; or were duplicate database entries having the same user ID eliminated before analysis? In the latter case, which entries were kept for analysis (eg, the first entry or the most recent)? | Unknown |
| IP check | Indicate whether the IP address of the client computer was used to identify potential duplicate entries from the same user. If so, mention the period of time for which no two entries from the same IP address were allowed (eg, 24 hours). Were duplicate entries avoided by preventing users with the same IP address access to the survey twice; or were duplicate database entries having the same IP address within a given period of time eliminated before analysis? If the latter, which entries were kept for analysis (eg, the first entry or the most recent)? | NA |
| Log file analysis | Indicate whether other techniques to analyze the log file for identification of multiple entries were used. If so, please describe. | NA |
| Registration | In “closed” (non-open) surveys, users need to login first and it is easier to prevent duplicate entries from the same user. Describe how this was done. For example, was the survey never displayed a second time once the user had filled it in, or was the username stored together with the survey results and later eliminated? If the latter, which entries were kept for analysis (eg, the first entry or the most recent)?  *Duplicate entries were avoided by preventing access to the survey if the LEO panelist was already registered in the LEO database as having completed the survey.* | Additional File 2 |
| Handling of incomplete questionnaires | Were only completed questionnaires analyzed? Were questionnaires which terminated early (where, for example, users did not go through all questionnaire pages) also analyzed?  *Of the 3,594 individuals who started the survey, 394 (11%) exited before completing it; the remaining 3,200 (89%) completed all questions and were included in the analysis.* | Page 3 |
| Questionnaires submitted with an atypical timestamp | Some investigators may measure the time people needed to fill in a questionnaire and exclude questionnaires that were submitted too soon. Specify the timeframe that was used as a cut-off point, and describe how this point was determined. | NA |
| Statistical correction | Indicate whether any methods such as weighting of items or propensity scores have been used to adjust for the non-representative sample; if so, please describe the methods.  *In our study we used the Random Interactive Method that permitted adjusting for multiple characteristics while keeping each proportionate as a whole; The weights for* *each age, sex, and region categories were calculated based on current census population distributions[28]; an overall correction for each province and territory was then applied.* | Page 3 |

Eysenbach G. Improving the quality of Web surveys: the Checklist for Reporting Results of Internet E-Surveys (CHERRIES). J Med Internet Res. 2004 Sep 29;6(3):e34 [erratum in J Med Internet Res. 2012; 14(1): e8.]. Article available at [https://www.jmir.org/2004/3/e34](https://www.jmir.org/2004/3/e34/)/; erratum available <https://www.jmir.org/2012/1/e8/>. Copyright ©Gunther Eysenbach. Originally published in the [Journal of Medical Internet](http://www.jmir.org) Research, 29.9.2004 and 04.01.2012.

This is an open-access article distributed under the terms of the Creative Commons Attribution License (<https://creativecommons.org/licenses/by/2.0/>), which permits unrestricted use, distribution, and reproduction in any medium, provided the original work, first published in the Journal of Medical Internet Research, is properly cited.
