# Supplementary material for: Public awareness and knowledge of sepsis: a cross-sectional survey of adults in Canada
Source: Crit Care. 2022 Nov 3;26:337. doi: 10.1186/s13054-022-04215-6 (PMC9632573; doi:10.1186/s13054-022-04215-6)
Supplement: Supplementary file 2 — Additional file 2. Sepsis Survey Development and Format [file 13054_2022_4215_MOESM2_ESM.docx]

**Additional File 2. Sepsis Questionnaire Development and Format**

The final questionnaire comprised of 28 questions measuring the three content domains—sepsis awareness (n=3), sepsis information and access (n=7), and sepsis knowledge (n=18)—and a 17-item demographics section (e.g., age, gender, ethnicity, education). We further categorized the 18 knowledge questions into four broad topics related to sepsis: (1) definition, (2) signs and symptoms, (3) mortality and risk factors, and (4) prevention. The content domains and corresponding topics are presented in the figure below. The English version of the questionnaire is subsequent.

Most questions were single-response multiple choice (i.e., select only one of many) or multiple-response multiple choice (i.e., select all that apply). We defined ‘Sepsis Awareness’ as a binary variable to the question “*Have you heard of the medical condition called sepsis*?” with ‘Yes’ coded as ‘Aware’, and ‘No’ or ‘Uncertain’ coded as “Unaware”. We included one 5-point unipolar scale (1= very poor, 5=very good) to measure respondent’s self-report of their knowledge of sepsis (“*How would you rate your level of knowledge about sepsis*?”) and two open-ended questions (“*Briefly describe in your own words ‘what is sepsis*?“ and “*Please list common symptoms or signs of sepsis*?”). All questions included a “don’t know” or “prefer not to answer” option. Duplicate entries were avoided by preventing access to the survey if the LEO panelist was already registered in the LEO database as having completed the survey.

The final questionnaire domains and topics are represented in the following figure.


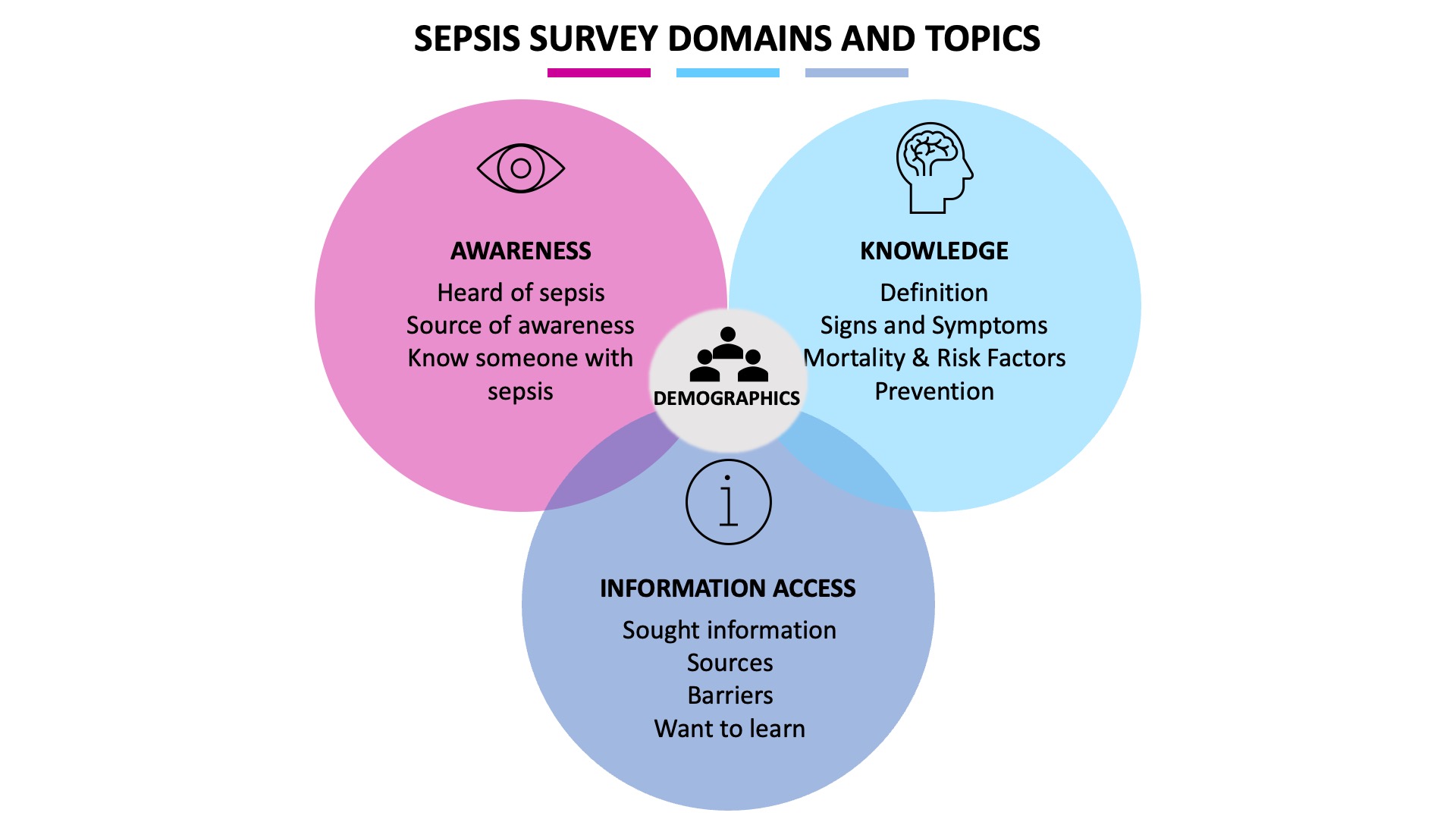


**Public Awareness and Understanding of Sepsis (English Questionnaire)**

**Dans quelle langue souhaitez-vous répondre à ce sondage? / In what language would you like to complete this survey?**

**O** Français

**O** English

[After language selection, survey begins with implied consent in the appropriate language then on to S1]

**SCREENING QUESTIONS FOR QUOTA MANAGEMENT**

**S1. How old are you?**

RESP_AGE

Single response

**[PN: THANK AND TERMINATE IF UNDER 18]**

**S2. What gender do you most identify with?**

**O** Woman/girl

**O** Man/boy

**O** Non-binary

**O** Two-spirited

**O** Prefer to self-describe: __________

**O** Prefer not to answer

**S3. Please indicate your sex?** Note: As indicated by Statistics Canada, transgender, transsexual, and intersex Canadians should indicate the sex (male or female) with which they most associate themselves.

**O** Female

**O** Male

**O** Prefer not to answer

**S4. What province or territory do you live in?**

**O** British Columbia

**O** Alberta

**O** Saskatchewan

**O** Manitoba

**O** Ontario

**O** Quebec

**O** Newfoundland and Labrador

**O** Prince Edward Island

**O** New Brunswick

**O** Nova Scotia

**O** Yukon

**O** Northwest Territories

**O** Nunavut

**MAIN QUESTIONNAIRE BODY**

We are interested in your awareness and understanding of sepsis. Please answer all the questions in this survey without checking the internet or other sources*.*

1. **Have you ever heard of the medical condition sepsis?**

**O** Yes

**O** No **[SKIP to Q9]**

**O** I don’t know/Uncertain **[SKIP to Q9]**

**[PN - DISPLAY IF RESPONSE TO QUESTION 1 “**Have you ever heard of the medical condition sepsis?**“ IS “Yes”.**

1. **How did you hear about sepsis? Please select all that apply.**

**O** I had sepsis

**O** A family member had sepsis

**O** A friend or coworker had sepsis

**O** A family member told me about sepsis

**O** A friend or co-worker told me about sepsis

**O** A healthcare provider (e.g., doctor, nurse) in a hospital told me about sepsis

**O** A healthcare provider (e.g., doctor, nurse) in a community clinic told me about sepsis

**O** I am a healthcare provider or work in healthcare

**O** From a newspaper or magazine (ad or article)

**O** A scientific journal article(s)

**O** Television (medical series, medical news, interviews with healthcare providers)

**O** The radio

**O** The internet (ad or website)

**O** Social media platforms (e.g., Facebook, Twitter, Instagram)

**O** An application downloaded on my cellphone or tablet

**O** School or an education session (e.g., lecture, workshop)

**O** Other (please specify): ________________

**O** I don’t know or remember how I heard about sepsis

1. **Have you ever actively looked for information about sepsis?**

**O** Yes

**O** No **[SKIP to Q9]**

**O** I don’t know/don’t remember **[SKIP to Q9]**

**[PN - DISPLAY THE FOLLOWING QUESTIONS IF RESPONSE TO QUESTION 3 “**Have you ever actively looked for information about sepsis?**“ IS “Yes”.**

1. **Where did you look for information about sepsis? Please select all that apply.**

**O** From a family member

**O** From a friend

**O** From a co-worker

**O** From a sepsis survivor

**O** From a healthcare provider (e.g., doctor, nurse)

**O** Television (ad or program)

**O** Newspapers / Magazines (ad or article)

**O** Scientific journals

**O** The Internet (via search engines or websites)

**O** Social media platforms (e.g., Facebook, Twitter, Instagram)

**O** An application downloaded on my cellphone or tablet

**O** From a teacher in school or attended a workshop or education session

**O** Other (please specify): ________________

**O** I don’t remember where I looked for information about sepsis

1. **Which of the following reasons MOST influenced where you looked for information about sepsis?** (choose only one option)

**O** Ease of access to information

**O** User friendly format

**O** Reliability of information

**O** Trust in information source

**O** Low cost

**O** Confidentiality of information

**O** Other (please name the reason): ___________________________________

**O** I don’t know

1. **Did you experience any difficulties getting the information you wanted or needed about sepsis?**

**O** Yes

**O** No **[SKIP to Q8 or Q9]**

**O** I don’t know **[SKIP to Q8 or Q9]**

**[PN - DISPLAY IF RESPONSE TO QUESTION 6 “**Did you experience any difficulties getting the information you needed about sepsis?**“ IS “Yes”.**

1. **Please select the difficulties that you experienced in getting the information you wanted or needed about sepsis.** (multiple responses allowed)

**O** Information was not explained in plain language

**O** Lack of information on the topic I was searching

**O** Lack of information available on demand (when I needed it)

**O** Difficulty in determining the quality of the information (i.e., whether the information was reliable)

**O** Other (please specify): ________________

**O** I don’t know

**[PN - DISPLAY IF RESPONSE TO QUESTION 4 “***Where did you look for information about sepsis?“*  **DID NOT INCLUDE “the Internet”]**

1. **What was your main reason for not using the internet to look for information about sepsis?** (choose only one option)

**O** I think that the information provided on the internet is not reliable

**O** I think that the information provided on the internet cannot be trusted

**O** I find it difficult to get the information I am looking for using the internet

**O** I did not have reliable access to the internet

**O** Other (please name the reasons(s)): _____

1. **How would you like to learn about sepsis? Please select all that apply.**

**O** From a family member

**O** From a friend

**O** From a co-worker

**O** From a sepsis survivor

**O** From a healthcare provider (e.g., doctor, nurse))

**O** Television (ad or program)

**O** Newspapers / Magazines (ad or article)

**O** Scientific journals

**O** The Internet (via search engines or websites)

**O** Social media platforms (e.g., Facebook, Twitter, Instagram)

**O** An application downloaded on my cellphone or tablet

**O** From a teacher in school or attend a workshop or education session

**O** Other (please specify): ________________

**O** I don’t know how I would like to learn about sepsis

**O** I would not like to learn about sepsis **[SINGLE SELECT]**

**[DISPLAY QUESTIONS TO ALL]**

1. **Do you know anyone who has ever had sepsis? Please select all that apply.**

**O** Myself

**O** My Spouse/partner

**O** My Child

**O** My Mother

**O** My Father

**O** My Sibling

**O** Other relative

**O** Friend

**O** Co-worker or acquaintance

**O** Other (please specify): ________________

**O** No, I do not personally know anyone who has had sepsis **[SINGLE SELECT]**

1. **How would you rate your level of knowledge about sepsis?**

| **O** | **O** | **O** | **O** | **O** |
| --- | --- | --- | --- | --- |
| Very Poor | Poor | Average | Good | Very Good |

**[PN: DISPLAY IF RESPONDENT ANSWERS “Yes” TO “**Have you ever heard of the medical condition sepsis**”, ASK the following open-ended questions]**

1. **Briefly describe in your own words “what is sepsis”. (1-3 sentences)**

|  |
| --- |

1. **Please list common symptoms or signs of sepsis.**

|  |
| --- |

**[DISPLAY TO ALL]**

1. **Which of the following statements about sepsis is true?**

**O** Sepsis is a severe allergic reaction

**O** Sepsis is a seizure involving violent muscle contractions

**O** Sepsis is the body’s extreme response to an infection

**O** I don’t know

1. **Which of the following statements about symptoms of sepsis is true?**

**O** Weakness or numbness on one side of the body is a common symptom of sepsis

**O** An infected wound with bloody pus is always a symptom of sepsis

**O** Sepsis is associated with a combination of symptoms (no single symptom indicates sepsis)

**O** I don’t know

1. **Select the word(s) or phrase(s) that describe sepsis.** (multiple answers allowed)

**O** Severe allergic reaction

**O** Poisoning by eating contaminated food

**O** Infection

**O** Inflammation

**O** The body’s extreme response to an infection

**O** None of the above responses describe sepsis **[SINGLE SELECT]**

**O** I don’t know

1. **Which of the following, if any, are common symptoms or signs of sepsis?** (multiple answers allowed)

**O** Fever

**O** Infection

**O** Feeling extremely ill (like you are going to die)

**O** Pain in left shoulder

**O** Slurred speech or confusion

**O** Indigestion

**O** Fast heart rate

**O** Passing no urine all day

**O** Fast breathing/severe breathlessness

**O** Weakness or numbness on one side of the body

**O** Extreme shivering or muscle pain

**O** Skin blotchy or discolored

**O** None of the above are common symptoms or signs of sepsis **[SINGLE SELECT]**

**O** I don’t know

1. **Sepsis is contagious.**

**O** True

**O** False

**O** I don’t know

1. **Sepsis is the leading cause of death worldwide compared to *all other* medical conditions.**

**O** True

**O** False

**O** I don’t know

1. **Roughly what percentage of deaths around the world are due to sepsis each year?**

**O** 5%

**O** 15%

**O** 20%

**O** 30%

**O** I don’t know

1. **Which of the following factors are associated with a higher risk of a person developing sepsis?** (multiple answers allowed).

**O** Age **[DISPLAY Q23]**

**O** Income level

**O** Sex **[DISPLAY Q22]**

**O** Education level

**O** Race/ethnicity

**O** Living in a shared housing facility (e.g., nursing home)

**O** Pre-existing medical conditions (e.g., diabetes)

**O** I don’t know

**[PN: IF RESPONDENT ANSWERS “Sex” TO “**Which of the following factors are associated with higher risk of developing sepsis?**”, ASK the following True/False question]**

1. **Males have a higher risk of developing sepsis than females.**

**O** True

**O** False

**O** I don’t know

**[PN: IF RESPONDENT ANSWERS “Age” TO “**Which of the following factors are associated with higher risk of developing sepsis?**”, ASK the following True/False question]**

1. **Young children (<5 years) and older adults (≥65 years) have a higher risk of developing sepsis than people in other age groups.**

**O** True

**O** False

**O** I don’t know

**[PN: IF RESPONDENT ANSWERS “Pre-existing conditions” TO “**Which of the following factors are associated with higher risk of developing sepsis?**”, ASK the following question]**

1. **Select the medical conditions that are associated with a higher risk of developing sepsis.** (multiple answers allowed)

**O** An infection (any bacterial, parasitic, viral, fungal infection)

**O** Weakened immune system

**O** Heart disease

**O** COVID-19

**O** Obesity

**O** Diabetes

**O** Stroke

**O** Cancer

**O** Influenza (the flu)

**O** None of the above medical conditions are associated with a higher risk of sepsis **[SINGLE SELECT]**

**O** I don’t know

**[DISPLAY TO ALL]**

1. **Which of the following actions, if any, can help prevent or lower your risk of developing sepsis?** (multiple answers allowed)

**O** Drinking lots of fluids

**O** Keeping vaccinations up-to-date (e.g., seasonal influenza (flu) shot, SARS-CoV-2 (COVID-19) shot)

**O** Getting 8 hours of sleep a night

**O** Hand washing

**O** Eating a balanced diet

**O** Personal hygiene (keeping your body clean)

**O** Treating infections

**O** None, sepsis cannot be prevented **[SINGLE SELECT]**

**O** I don’t know

**[PN: IF RESPONDENT DOES NOT ANSWER “Vaccination” TO “**Which of the following actions, if any, can help prevent or lower your risk of developing sepsis**”, ASK the following questions]**

1. **Vaccination for seasonal influenza (i.e., the flu) can decrease my risk of developing sepsis?**

**O** True

**O** False

**O** I don’t know

1. **Vaccination for SARS-CoV2 (i.e., COVID-19) can decrease my risk of developing sepsis?**

**O** True

**O** False

**O** I don’t know

**[PN: IF RESPONDENT ANSWERS “Myself or My spouse/partner or My child or My mother or My father or My sibling or My sibling or Other relative” TO** “Do you know anyone who has ever had sepsis**”, ASK the following question]**

1. **Which of the following problems are common among sepsis survivors?** (multiple answers allowed)

O Difficulty getting to or staying asleep including nightmares

O Feeling anxious, worried, or having panic attacks

O Being depressed, unmotivated, and have decreased cognitive function

O Severe muscle and joint pains

O Dry and itchy skin with brittle nails and hair loss

O Organ dysfunction (kidney failure, lung problems, etc.)

O None of the above problems are common among sepsis survivor **[SINGLE SELECT]**

O I don’t know

**[[PN: IF RESPONDENT ANSWERS “Myself or My spouse/partner or My child or My mother or My father or My Sibling” TO** “Do you know anyone who has ever had sepsis**”, ASK the following question]**

1. **When you or someone close to you became ill with sepsis, where did you or the person close to you seek medical support? Please select all that apply.**

O A community or walk-in clinic

O An urgent care or hospital emergency department

O A telephone health advice line (e.g., Healthlink, Telecare, Info-Santé)

O 911 or emergency line (for ambulance)

O The Internet (e.g., searched symptoms)

O Social media (e.g., asked a question)

O A relative or friend for advice

O Other (please specify): ________________

O I don’t know or don’t remember

**[PN: IF RESPONDENT ANSWER “Other relative or Friend or Co-worker or Acquaintance or Other or No,** I **do not personally know anyone who has had sepsis ” TO** “Do you know anyone who has ever had sepsis**”, ASK the following questions]**

1. **If you or someone close to you becomes ill with symptoms of sepsis, where would you seek medical support? Please select all that apply.**

O A community or walk-in clinic

O An urgent care or hospital emergency department

O A telephone health advice line (e.g., Healthlink, Telecare, Info-Santé)

O 911 or emergency line (for ambulance)

O The Internet (e.g., search for symptoms)

O Social media (e.g., ask a question)

O A relative or friend

O Other (please specify): ________________

**[READ SCREEN FOR ALL]**

**DEMOGRAPHIC SECTION**

**Thank you. We would now like to ask you some demographic questions to help us categorize the responses. As a reminder, your answers will be anonymous and will only be used in grouped data analysis.**

**D1. What is your current marital status?**

**O** Single, never married

**O** Separated or divorced (in process or finalized)

**O** Married

**O** Living with partner

**O** In a relationship, but not living together

**O** Widowed

**O** Prefer not to answer

**D2. What is the size of the town or city you live in?**

**O** Small town or city (up to 10,000 people)

**O** Medium sized city (over 10,000 people, up to 100,000 people)

**O** Large city (over 100,000 people, up to 1,000,000 people)

**O** Large metropolitan area (over 1,000,000 people)

**O** I don’t know

**O** Prefer not to answer

**D3. How many people live in your household, including yourself?**

*(Please include all other family members and / or roommates living in the same house)*

___ person / people [verification: integer]

**O** Myself **[SKIP to D5]**

**O** Prefer not to answer **[SKIP to D5]**

**D4. Are there any children who are UNDER 18 YEARS OF AGE living in your household?**

**O** Yes

**O** No, there are no children under 18 years old living in my household

**O** I prefer not to answer

**D5. What is your current employment status? Please select all that apply.**

**O** Employed (working full-time hours)

**O** Employed (working part-time/casual hours)

**O** Retired **[SKIP to D7]**

**O** Student (full or part-time) **[SKIP to D7]**

**O** Full time parent or homemaker **[SKIP to D7]**

**O** Currently unemployed or unable to work for any reason (including laid off) **[SKIP to D7]**

**O** Other (please specify): ________________ **[SKIP to D7]**

**O** Prefer not to answer **[SKIP to D7]**

**D6. What is the MAIN employment sector that you work in? Examples are included. Please select the most appropriate categories.**

**O** Animal Careers
**O** Aviation
**O** Arts
**O** Business
**O** Education
**O** Law Enforcement
**O** Media
**O** Medical/Health

**O** Military Careers
**O** Service Industry

**O** Science, Technology, Engineering, and Math (STEM) Careers

**O** Other (please specify): ________________

**O** Prefer not to answer

**D7. What was your total overall household income in 2020 before taxes?**

**O** $19,999 or less

**O** Between $20,000 and $39,999

**O** Between $40,000 to $59,999

**O** Between $60,000 to $79,999

**O** Between $80,000 to $99,999

**O** Between $100,000 to $124,999

**O** Between $125,000 and $149,000

**O** $150,000 or more

**O** I don’t know

**O** Prefer not to answer

**D8. Which ethnic, racial, or cultural group do you most closely self-identify with? (please select all that apply)** Please note that the examples provided are non-exhaustive and are meant to be a guide to help you respond to the question.

O Asian - East (e.g., Chinese, Japanese, Korean)

O Asian - South (e.g., Indian, Pakistani, Sri Lankan)

O Asian - South East (e.g., Malaysian, Filipino, Vietnamese)

O Black - African (e.g., Ghanaian, Kenyan, Somali)

O Black - Caribbean (e.g., Barbadian, Jamaican)

O Black - North American (e.g., Canadian, American)

O First Nations

O Indian - Caribbean

O Indigenous

O Inuit

O Latin American (e.g., Argentinean, Chilean, Salvadoran)

O Metis

O Middle Eastern (e.g., Egyptian, Iranian, Lebanese)

O White - European (e.g., English, Italian, Portuguese, Russian)

O White - North American (e.g., Canadian, American)

O Mixed Heritage (please specify): ________________________________________________

O Other ethnic, racial, or cultural group (please specify): ____________________________________

O I don’t know

O Prefer not to answer

**D9. Which of the following best describes your faith / religious identity?**

**O** Roman Catholic

**O** Protestant or other Christian

**O** Muslim

**O** Jewish

**O** Hindu

**O** Sikh

**O** Other (please specify): ________________

**O** No Religious Identity

**O** Don't know

**O** Prefer not to answer

**D10. What is the highest level of education you have completed?**

**O** Less than a high school diploma

**O** High school diploma

**O** Collège d'enseignement general et professionnel (CEGEP)

**O** Vocational college

**O** Trade certification

**O** Some college (no degree)

**O** College degree

**O** Some university (no degree)

**O** Undergraduate degree (Bachelor’s)

**O** Graduate degree (Masters or Doctorate)

**O** Professional degree (MD, JD, DDS, etc.)

**O** Other (please specify): ________________

**O** Prefer not to answer

**D11. How long have you been in Canada?**

O Less than 1 year

O 1 year to 4 years

O 5 years to 10 years

O 11 years to 20 years

O 21 years or more

O I don’t know

O I prefer not to answer

**D12. Are you currently diagnosed with any of the following health conditions? Please select all that apply.** *Please do not select any conditions you may have been previously diagnosed but no longer have.*

**O** Autoimmune disease (e.g. lupus, psoriasis, rheumatoid arthritis, Crohn’s disease, etc.)

**O** Cancer

**O** Diabetes (Type 1 or Type 2)

**O** Cardiovascular disease (e.g. arrhythmias, coronary artery diseases, hypertension)

**O** Obesity

**O** Other chronic diseases (e.g. high cholesterol, kidney disease, osteoarthritis, neuromuscular, etc.)

**O** Other (please specify): ________________

**O** I am not currently diagnosed with any of these health conditions **[SKIP to D14]**

**O** Prefer not to answer **[SKIP to D14]**

**D13. Do you think the health condition(s) you selected is/are a risk factor for Sepsis?**

**O** Yes

**O** No

**O** I don’t know

**D14. Have you recently had a severe illness or been hospitalized?**

**O** Yes

**O** No

**O** Prefer not to answer

**[READ SCREEN FOR ALL]**

From time to time, our research team asks people if they would be willing to be contacted to participate in follow up research with us that is related to the survey topic. This may involve participating in interviews or focus groups, either in person or online. In focus groups, we gather a group of people to talk about issues of interest to our research topic. There may also be an incentive like a gift card offered for participating.

If you are interested in being contacted about future studies, we will ask you to provide your first name and contact information. By agreeing to be contacted, you are consenting for a member of our research team to email you with details about other studies we are conducting. Your name will be added to a list of contacts for future participation for up to 1 year. This does not guarantee that you will be contacted. If you are contacted about a study, you are free to choose to participate or not participate. The contact list will be saved in a password protected file on an encrypted server at the University of Calgary. Only members of our research team will have access to this list. You may withdraw your consent to be contacted by contacting the study team at [C3ResNetwork@ucalgary.ca](mailto:C3ResNetwork@ucalgary.ca). Your contact information **will not be linked at all** to your current survey responses.

As per Leger’s data storage and transfer protocols, your personal contact information will be initially collected on Leger’s data server in Montreal before being transferred to our study team.

**D15. Are you interested in being contacted about follow up research conducted by our research team on topics related to sepsis awareness and information-seeking?**

**O** Yes, I consent to provide my contact information to learn about future research studies

**O** No, I do not wish to be contacted to learn about future studies.

**[IF D15 = “I CONSENT”, ASK D16 and D17, ELSE SKIP TO CLOSE]**

**D16. Thank you for your interest. Please provide an email address where a member of our research team can contact you:**

[RECORD EMAIL ADDRESS. ENSURE VALID EMAIL FORMAT.]

**D17. Could we please have your first name?**

[RECORD FIRST NAME

Thank you for participating in our survey!

**SUBMIT**

**[READ SCREEN – END OF SURVEY MESSAGE]**

**
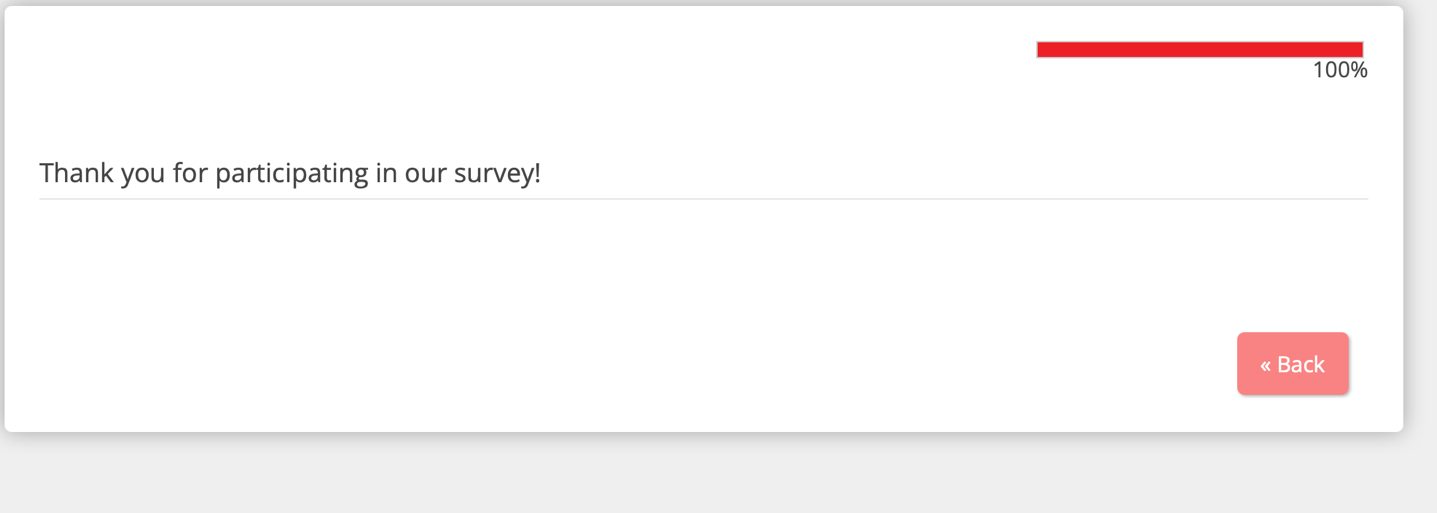
**

Sepsis is not an infection but occurs when your immune system responds in the wrong way to an infection. This causes inflammation throughout your body which can quickly lead to organ failure and death.  Sepsis is a medical emergency!  Early recognition is the key to prevention.  Please visit [www.sepsisawareness.ca](http://www.sepsisawareness.ca) for more information about sepsis.

**September is Sepsis Awareness Month | September 13^th^ is World Sepsis Day**
