# Supplementary material for: Public awareness and knowledge of sepsis: a cross-sectional survey of adults in Canada
Source: Crit Care. 2022 Nov 3;26:337. doi: 10.1186/s13054-022-04215-6 (PMC9632573; doi:10.1186/s13054-022-04215-6)
Supplement: Supplementary file 3 — Additional file 3. Sepsis Knowledge Question Scoring Key [file 13054_2022_4215_MOESM3_ESM.docx]

**Additional File 3. Sepsis Knowledge Question Scoring Key**

We calculated composite knowledge scores for each respondent based on correct identification of response options to each knowledge question. Correctly selected responses options (in green text) were coded as 1; incorrect and “don’t know” response options were coded as 0 (i.e., respondents were not penalized for selecting incorrect answers). The total number of possible correct answers was 26. We converted the score to a percent for each respondent (i.e., number of correctly selected options (n=x) divided by total possible correct answers (n=26) x 100).

| **Domain** | **Question** | **Score** |
| --- | --- | --- |
| Definition | **Which of the following statements about sepsis is true?** | |
|  | Sepsis is a severe allergic reaction |  |
|  | Sepsis is a seizure involving violent muscle contractions |  |
|  | Sepsis is the body’s extreme response to an infection | 1 |
|  | I don’t know |  |
| Signs & Symptoms | **Which of the following statements about symptoms of sepsis is true?** | |
|  | Weakness or numbness on one side of the body is a common symptom of sepsis |  |
|  | An infected wound with bloody pus is always a symptom of sepsis (2) |  |
|  | Sepsis is associated with a combination of symptoms (no single symptom indicates sepsis) | 1 |
|  | I don’t know |  |
| Definition | **Select the word(s) or phrase(s) that describe sepsis.** | |
|  | Allergic reaction |  |
|  | Poisoning by eating contaminated food |  |
|  | Infection | 1 |
|  | Inflammation | 1 |
|  | The body’s extreme response to an infection | 1 |
|  | None of the above responses describe sepsis |  |
|  | I don’t know |  |
| Signs & Symptoms | **Which of the following, if any, are common symptoms or signs of sepsis** | |
|  | Fever | 1 |
|  | Infection | 1 |
|  | Feeling extremely ill (like you are going to die) | 1 |
|  | Pain in left shoulder |  |
|  | Slurred speech or confusion | 1 |
|  | Indigestion |  |
|  | Fast heart rate | 1 |
|  | Passing no urine all day | 1 |
|  | Fast breathing/severe breathlessness | 1 |
|  | Weakness or numbness on one side of the body |  |
|  | Extreme shivering or muscle pain | 1 |
|  | Skin blotchy or discolored | 1 |
|  | None of the above are common symptoms or signs of sepsis |  |
|  | I don’t know |  |
| Definition | **Sepsis is contagious.** | |
|  | True |  |
|  | False | 1 |
|  | I don’t know |  |
| Mortality & Risk Factors | **Sepsis is the leading cause of death worldwide compared to *all other* medical conditions.** | |
|  | True | 1 |
|  | False |  |
|  | I don’t know |  |
|  | **Roughly what percentage of deaths around the world are due to sepsis each year?** | |
|  | 5% |  |
|  | 15% |  |
|  | 20% | 1 |
|  | 30% |  |
|  | I don’t know |  |
|  | **Which of the following factors are associated with a higher risk of a person developing sepsis?** | |
|  | Age | 1 |
|  | Income level |  |
|  | Sex | 1 |
|  | Education level |  |
|  | Race/ethnicity | 1 |
|  | Living in a shared housing facility (e.g., nursing home) | 1 |
|  | Pre-existing medical conditions (e.g., diabetes) | 1 |
|  | I don’t know |  |
| Prevention | **Which of the following actions, if any, can help prevent or lower your risk of developing sepsis?** | |
|  | Drinking lots of fluids |  |
|  | Keeping vaccinations up to date (e.g., seasonal influenza (flu) shot, SARS-CoV-2 (COVID-19) shot) | 1 |
|  | Getting 8 hours of sleep a night |  |
|  | Hand washing | 1 |
|  | Eating a balanced diet |  |
|  | Personal hygiene (keeping your body clean) | 1 |
|  | Treating infections | 1 |
|  | None, sepsis cannot be prevented |  |
|  | I don’t know |  |

| **TOTAL SCORE** | **26** |
| --- | --- |
| Definition | 5 |
| Signs/Symptoms | 10 |
| Mortality & Risk Factors | 7 |
| Prevention | 4 |
