# Supplementary material for: Public awareness and knowledge of sepsis: a cross-sectional survey of adults in Canada
Source: Crit Care. 2022 Nov 3;26:337. doi: 10.1186/s13054-022-04215-6 (PMC9632573; doi:10.1186/s13054-022-04215-6)
Supplement: Supplementary file 4 — Additional file 4. Awareness of Sepsis Additional Results. Table S1. Distribution of responses for items related to awareness of sepsis. Figure S1. Regional differences in awareness of sepsis and interest in learning about sepsis [file 13054_2022_4215_MOESM4_ESM.docx]

**Additional File 4. Awareness of Sepsis**

Table of Contents

[Table S1. Distribution of responses for items related to awareness of sepsis 2](#_Toc114833268)

[Figure S1. Regional differences in awareness of sepsis and interest in learning about sepsis 4](#_Toc114833269)

# Table S1. Distribution of responses for items related to awareness of sepsis

| **Questionnaire Items** |  | Weighted Percent | | |
| --- | --- | --- | --- | --- |
|  | Total n | Yes | No | Don’t know |
| ***Heard of the term sepsis^1^*** | 3200 | 61.4 | 32.6 | 6.0 |
| Source where heard about sepsis^2^ | 1978^3^ | 82.2 | - | 17.8 |
| **Traditional Media** | 1626^4^ | **41.9** | **58.1** | *-* |
| Television |  | 27.7 | 72.3 | - |
| Newspaper or magazine |  | 15.0 | 85.0 | - |
| Radio |  | 2.0 | 98.0 | - |
| **Someone told me about sepsis** |  | **23.1** | **76.9** | **-** |
| Family member |  | 7.0 | 93.0 | - |
| HCP in hospital |  | 7.7 | 92.3 | - |
| HCP in community |  | 2.7 | 97.3 | - |
| Friend/Coworker |  | 7.0 | 93.0 | - |
| **Someone I knew had sepsis** |  | **23.9** | **76.1** | **-** |
| Family member |  | 11.2 | 88.8 | - |
| Friend or coworker |  | 5.8 | 94.2 | - |
| I had sepsis |  | 3.0 | 97.0 | - |
| **Academic** |  | **15.6** | **84.4** | **-** |
| School or education session |  | 7.9 | 92.1 | - |
| Scientific article |  | 6.2 | 93.8 | - |
| **Digital Media** |  | **14.6** | **85.4** | **-** |
| Internet |  | 8.7 | 91.3 | - |
| Social media |  | 4.1 | 95.9 | - |
| Computer application |  | 0.4 | 99.6 | - |
| I work in healthcare |  | 10.3 | 89.7 | - |
| Other |  | 6.1 | 93.9 | - |
| ***Knew someone who had sepsis****^5^* | 3200 | 21.2 | 78.8 | - |
| Myself | 690^6^ | 12.3 | 87.7 |  |
| **Family Member** |  | **46.2** | **53.8** |  |
| Spouse/partner |  | 7.4 | 92.6 |  |
| Child |  | 3.1 | 96.9 |  |
| Father |  | 6.4 | 93.6 |  |
| Mother |  | 5.9 | 94.1 |  |
| Sibling |  | 4.5 | 95.5 |  |
| Other relative |  | 21.9 | 78.1 |  |
| **Friend or co-worker** |  | **36.1** | **63.9** |  |
| Friend |  | 21.1 | 78.9 |  |
| Coworker |  | 16.8 | 83.2 |  |
| Other (patient, friend’s relative) |  | 15.5 | 84.5 |  |
| ***Would like to learn about sepsis****^7^* | 3200 | 94.3 | 5.7 | - |
| Preferred sources to learn from | 3014^8^ | 90.2 | - | 9.2 |
| Healthcare Provider | 2724 | 53.3 | 46.7 |  |
| Sepsis Survivor |  | 13.9 | 86.1 |  |
| **Digital Media** |  | **50.7** | **49.3** |  |
| Internet |  | 40.3 | 59.7 |  |
| Social media |  | 6.1 | 93.9 |  |
| Computer application |  | 2.2 | 97.8 |  |
| **Academic** |  | **35.1** | **64.9** |  |
| School or education session |  | 9.5 | 90.5 |  |
| Scientific article |  | 25.2 | 74.8 |  |
| **Traditional Media** |  | **23.0** | **77.0** |  |
| Television |  | 13.9 | 86.1 |  |
| Newspaper or magazine |  | 11.5 | 88.5 |  |
| **Family, friend, co-worker** |  | **14.6** | **85.3** |  |
| Family member |  | 9.2 | 90.8 |  |
| Friend |  | 6.5 | 93.5 |  |
| Co-worker |  | 3.1 | 96.9 |  |
| Other |  | 1.2 | 98.8 |  |

^1^ ‘Heard of the term sepsis’ was identified by the question *“Have you ever heard of the medical condition sepsis*?”, which included the response options ‘Yes’, ‘No’, and ‘Uncertain’.

^2^ Sources were collapsed to create composite variables as follows: ‘*Traditional Media*’ is combined from response selection to newspapers, television, and/or radio; ‘*Someone I knew had sepsis*’ is combined from responses to ‘A family member had sepsis’ and/or ‘A friend or coworker had sepsis’; ‘*Digital Media*’ is combined from responses to internet, social media, and computer/tablet application.

^3^ n equals the number of respondents who answered yes to the question “*Have you heard of the medical condition called sepsis?*”

^4^ n equals the number of respondents who identified at least one source where they heard about sepsis

^5^ ‘Knew someone with sepsis’ was identified by the question “*Do you know anyone who has ever had sepsis*?”, with ‘yes’ coded as ‘No’ to the response option “No, I do not personally now anyone who has ever had sepsis.”

^6^ n equals the number of respondents who identified ever knowing at least one person with sepsis

^7^ ‘Would like to learn about sepsis’ was identified by the question “*How would you like to learn about sepsis*?”, with ‘No’ coded to ‘I would not like to learn about sepsis’, ‘Don’t know’ coded to “I don’t know how I would like to learn about sepsis.”, and ‘Yes’ to all other response options.

^8^ n equals the number of respondents who identified at least one preferred source to learn about sepsis

Abbreviation: app=computer/tablet application

# Figure S1. Regional differences in awareness of sepsis and interest in learning about sepsis

‘Heard of sepsis’ was identified by the question *“Have you ever heard of the medical condition sepsis*?” coded as ‘Yes’. Respondents who did not select ‘No, I do not personally know anyone who has sepsis’ to the question ‘*Do you know anyone who has ever had sepsis*?” were coded as ‘Knows someone with sepsis’. Respondents who did not select ‘I would not like to learn about sepsis’ to the question “*How would you like to learn about sepsis*?” were coded as “Wants to learn about sepsis”. *p-value≤0.05, ***p-value≤0.001

Abbreviations: AB, Alberta; BC, British Columbia, MB, Manitoba; ON, Ontario; QC, Quebec; Atlantic, includes the provinces of New Brunswick, Newfoundland and Labrador, Nova Scotia, and Prince Edward Island; Territories, includes Northwest Territories, Nunavut, and Yukon.
