# Supplementary material for: Public awareness and knowledge of sepsis: a cross-sectional survey of adults in Canada
Source: Crit Care. 2022 Nov 3;26:337. doi: 10.1186/s13054-022-04215-6 (PMC9632573; doi:10.1186/s13054-022-04215-6)
Supplement: Supplementary file 5 — Additional file 5. Knowledge of Sepsis Additional Results. Figure S1. Sepsis awareness, perceived knowledge, and evaluated knowledge by respondent characteristics. Table S1. Distribution of responses for items related to knowledge of sepsis. Figure S2. Distribution of responses seeking medical support when ill with symptoms of sepsis [file 13054_2022_4215_MOESM5_ESM.docx]

**Additional File 5. Knowledge of Sepsis**

Table of Contents

[Figure S1. Sepsis awareness, perceived knowledge, and evaluated knowledge by respondent characteristics 2](#_Toc114834768)

[Table S1. Distribution of responses for items related to knowledge of sepsis 3](#_Toc114834769)

[Figure S2. Distribution of responses where to seek medical support when self or family member ill with symptoms of sepsis 5](#_Toc114834770)

Figure S1. Sepsis awareness, perceived knowledge, and evaluated knowledge by respondent characteristics
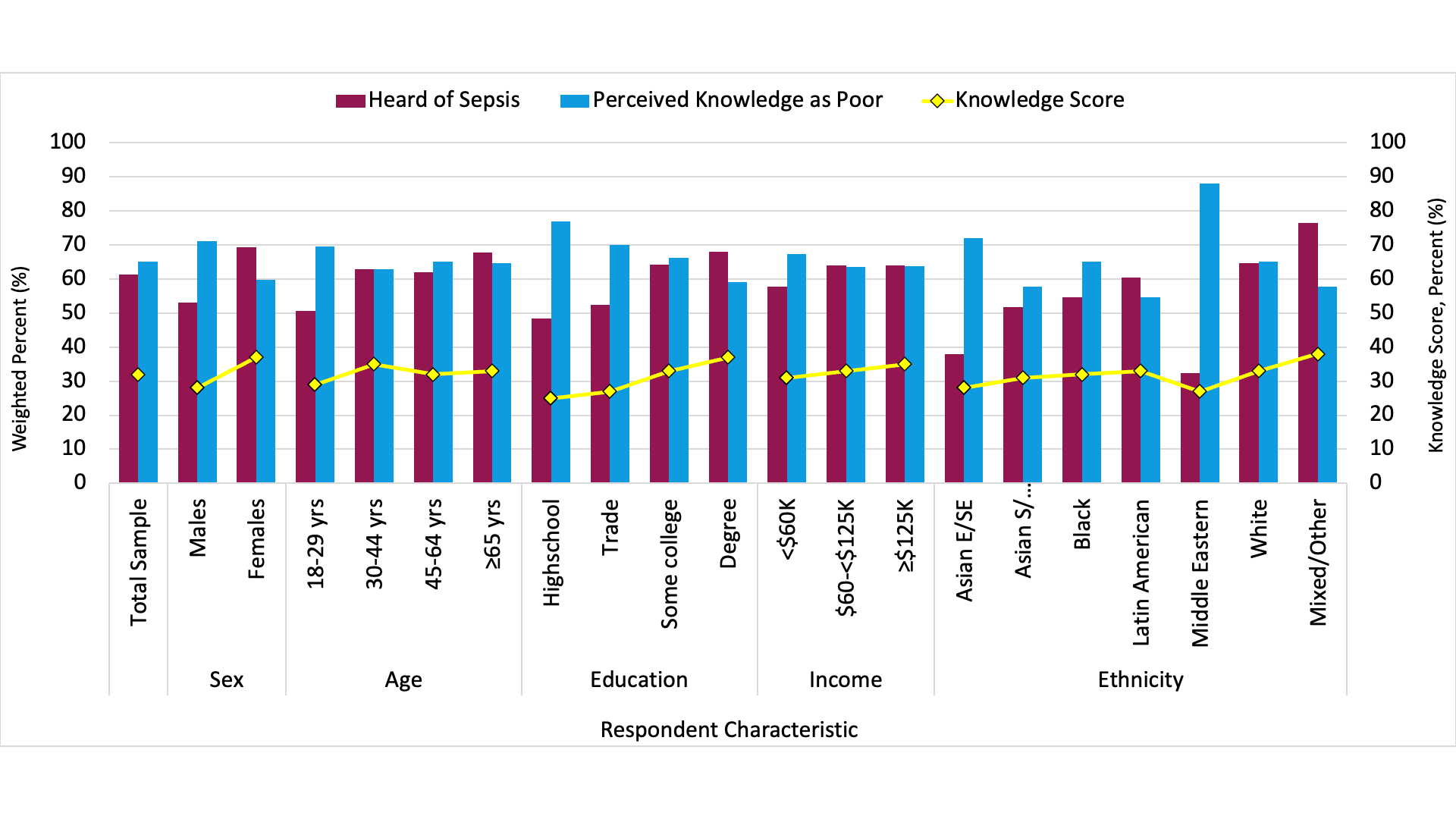
Heard of sepsis’ was coded as a binary variable to the question “*Have you heard of the medical condition called sepsis*?” with ‘Yes’ coded as ‘Heard of sepsis’, and ‘No’ or ‘Uncertain’ coded as “Not heard of sepsis”. Perceived level of knowledge was defined by a 5-point Likert scale question *“How would you rate your level of knowledge about sepsis*?” (1=very poor, 2=poor, 3=average, 4=good, 5=very good), with ‘Very Poor’ and ‘Poor’ collapsed into a single category, ‘Poor’. Mean Total Knowledge score was calculated by adding each respondents correct identification of response options to each knowledge question. Correctly selected responses options were coded as 1; incorrect and “don’t know” response options were coded as 0. The total number of possible correct answers was 26. The mean score for each respondent was converted to a percent ranging from 0 to 100.

# Table S1. Distribution of responses for items related to knowledge of sepsis

| **Questionnaire Items** |  | Weighted Percent | | |
| --- | --- | --- | --- | --- |
|  | Answer Key | Selected | Unselected | Don’t know |
| ***Total Knowledge*** (M=32.3%, SD=24.2%) |  |  |  |  |
| ***Definition*** (M=53.0%; SD=36.3%) |  |  |  |  |
| Sepsis is…. |  | - | - | 29.5 |
| ... a severe allergic reaction | I | 2.1 | 97.9 | - |
| … a seizure involving violent muscle contraction | I | 2.7 | 97.3 | - |
| … body’s extreme response to an infection | C | 65.7 | 34.3 | - |
| Sepsis is contagious (False) | C | 61.3 | 4.9 | 33.8 |
| Select the word(s) or phrase(s) that describe sepsis |  | - | - | 27.4 |
| Severe allergic reaction | I | 5.6 | 94.4 | - |
| Poisoning by eating contaminated food | I | 2.9 | 97.1 | - |
| Infection | C | 51.0 | 49.0 | - |
| Inflammation | C | 23.8 | 76.2 | - |
| The body’s extreme response to an infection | C | 61.3 | 38.7 |  |
| Selected at least one correct word or phrase |  |  |  | - |
| Selected all correct words or phrases |  |  |  | - |
|  |  |  |  |  |
| ***Signs and Symptom*** (M=31.5; SD=28.5) |  |  |  |  |
| Which of the following statements is true |  | - | - | 37.0 |
| Weakness or numbness on one side of the body is a common symptom of sepsis. | I | 2.5 | 97.5 | - |
| An infected wound with bloody pus is always a symptom of sepsis. | I | 10.0 | 90.0 | - |
| Sepsis is associated with a combination of symptoms (no single symptom indicates sepsis). | C | 50.6 | 49.4 | - |
| Which are common symptoms or signs of sepsis |  | - | - | 33.3 |
| Fever | C | 55.7 | 44.3 |  |
| Infection | C | 52.9 | 47.1 |  |
| Feeling extremely ill (like you are going to die) | C | 39.3 | 60.7 |  |
| Pain in left shoulder | I | 2.3 | 97.7 |  |
| Slurred speech or confusion | C | 12.5 | 87.5 |  |
| Indigestion | I | 3.9 | 96.1 |  |
| Fast heart rate | C | 26.4 | 73.6 |  |
| Passing no urine all day | C | 8.0 | 92.0 |  |
| Fast breathing/severe breathlessness | C | 21.2 | 78.8 |  |
| Weakness or numbness on one side of the body | I | 6.3 | 93.7 |  |
| Extreme shivering or muscle pain | C | 27.6 | 72.4 |  |
| Skin blotchy or discolored | C | 20.7 | 79.3 |  |
|  |  |  |  |  |
| ***Mortality and Risk Factors*** (M=16.5; SD=18.6) |  |  |  |  |
| Sepsis is the leading cause of death worldwide compared to all other medical conditions (True) | C | 8.8 | 33.4 | 57.9 |
| Percentage of global deaths due to sepsis each year |  | - | - | 62.9 |
| 5% | I | 13.4 | 86.6 | - |
| 15% | I | 13.1 | 86.9 | - |
| 20% | C | 7.7 | 92.3 | - |
| 30% | I | 2.9 | 97.1 | - |
| Factors related to higher risk of developing sepsis |  | - | - | 43.6 |
| Age | C | 28.2 | 71.8 | - |
| Income level | I | 8.1 | 91.9 | - |
| Sex | C | 4.2 | 95.8 | - |
| Education level | I | 4.3 | 95.7 | - |
| Race/ethnicity | C | 5.8 | 94.2 | - |
| Living in a shared housing facility | C | 13.6 | 86.4 | - |
| Pre-existing medical conditions | C | 47.8 | 52.2 | - |
|  |  |  |  |  |
| ***Prevention*** *(M=36.3, SD=37.1)* |  |  |  |  |
| Actions to prevent or lower risk of developing sepsis |  | - | - | 34.7 |
| Drinking lots of fluids | I | 18.9 | 81.1 | - |
| Keeping vaccinations up to date | C | 25.1 | 74.9 | - |
| Getting 8 hours of sleep a night | I | 10.8 | 89.2 | - |
| Hand washing | C | 29.8 | 70.2 | - |
| Eating a balanced diet | I | 14.7 | 85.3 | - |
| Personal hygiene (keeping your body clean) | C | 35.1 | 64.9 | - |
| Treating infections | C | 54.6 | 45.4 | - |
| None, sepsis cannot be prevented | I | 3.4 | 96.6 | - |

Abbreviations: C, Correct Answer; I, Incorrect answer

# Figure S2. Distribution of responses where to seek medical support when self or family member ill with symptoms of sepsis


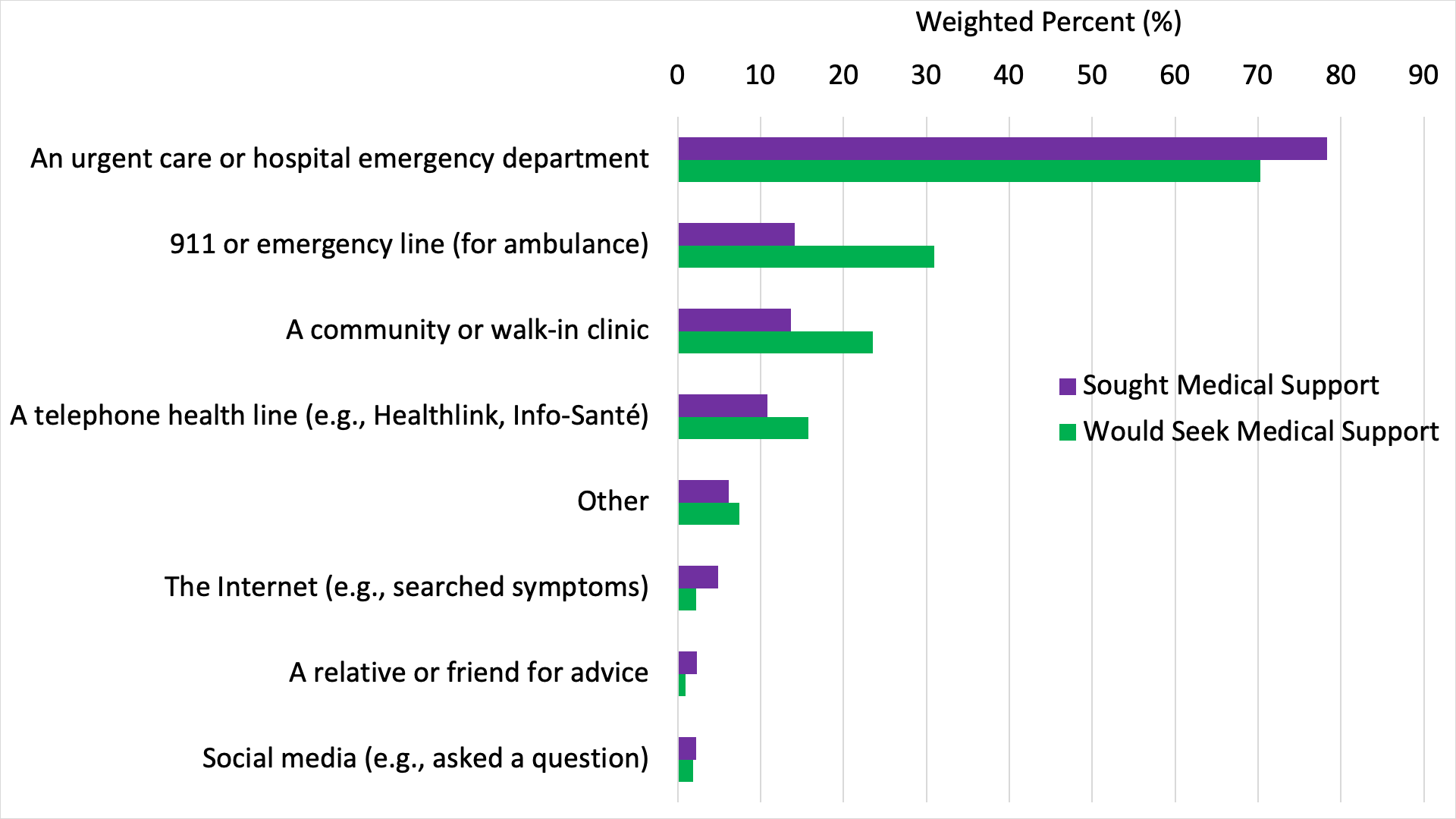


“Sought Medical Support’ includes respondents who indicated to the question ‘*Do you know anyone who has ever had sepsis?*’ either self or spouse/partner or child, or mother, or father, or sibling (n=249) and were displayed the question ‘*When you or someone close to you became ill with sepsis, where did you or the person close to you seek medical support?*’. ‘Would Seek Medical Support” includes respondents who indicated to the question ‘*Do you know anyone who has ever had sepsis?*’ either other relative or friend or co-worker or acquaintance or other or No, I do not personally know anyone who has had sepsis (n=2,951), and were displayed the question ‘*If you or someone close to you becomes ill with symptoms of sepsis, where would you seek medical support?*’
