# Supplementary material for: Public awareness and knowledge of sepsis: a cross-sectional survey of adults in Canada
Source: Crit Care. 2022 Nov 3;26:337. doi: 10.1186/s13054-022-04215-6 (PMC9632573; doi:10.1186/s13054-022-04215-6)
Supplement: Supplementary file 6 — Additional file 6. Distribution of responses for items related to sepsis information access [file 13054_2022_4215_MOESM6_ESM.docx]

**Additional File 6. Distribution of responses for items related to sepsis information access**

| **Questionnaire Items** |  | Weighted Percent | | |
| --- | --- | --- | --- | --- |
|  | Total n | Yes | No | Don’t know |
| ***Actively looked for information about sepsis*** | 1978***^1^*** | 19.7 | 76.9 | 3.4 |
| Sources used | 390^2^ |  |  |  |
| **From a person** |  |  |  |  |
| Family member |  | 11.7 | 88.3 | *-* |
| Friend |  | 8.8 | 91.2 | *-* |
| Co-worker |  | 8.9 | 91.1 | *-* |
| Sepsis survivor |  | 5.2 | 94.8 | *-* |
| Healthcare provider |  | 29.6 | 70.4 | *-* |
| **Traditional Media** |  |  |  |  |
| Television |  | 4.8 | 95.2 | *-* |
| Newspaper |  | 4.5 | 95.5 | *-* |
| **Digital Media** |  |  |  |  |
| Internet |  | 68.5 | 31.5 | *-* |
| Social media |  | 5.4 | 94.6 | *-* |
| Digital application |  | 3.4 | 96.6 | *-* |
| **Academic** |  |  |  |  |
| School or education session |  | 9.5 | 90.5 |  |
| Scientific articles |  | 24.9 | 75.1 |  |
| Other |  | 5.7 | 94.3 | *-* |
| Don’t know or remember |  | 0.8 | 99.2 | *-* |
| Reason that most influenced sources used |  | **-** | **-** | 2.0 |
| Ease of access to information |  | 37.1 | 62.9 | ***-*** |
| User friendly format |  | 7.9 | 92.1 | ***-*** |
| Reliability of information |  | 27.5 | 72.5 | ***-*** |
| Trust in information source |  | 22.1 | 77.9 | ***-*** |
| Low cost |  | 1.8 | 98.2 | ***-*** |
| Confidentiality of information |  | 0.6 | 99.4 | ***-*** |
| Other |  | 1.0 | 99 | ***-*** |
| Had difficulties getting information wanted | 34^3^ | 9.6 | 88.1 | 2.3 |
| Information was not in plain language |  | 45.3 | 54.7 | - |
| Lack of information on desired topic |  | 57.2 | 42.8 | - |
| Lack of information when needed |  | 16.5 | 83.5 | - |
| Difficulty in determining the quality of the information (i.e., if information is reliable) |  | 30.9 | 69.1 | - |
| Main reason for not using the internet | 118^4^ | 31.5 | 68.5 | - |
| Information is not reliable |  | 27.4 | 72.6 | - |
| Information cannot be trusted |  | 12.1 | 87.9 | - |
| Difficult to get the information looking for |  | 4.1 | 95.9 | - |
| No reliable access to the internet |  | 25.0 | 75.0 | - |
| Other |  | 31.5 | 68.5 | - |

1 n equals the number of respondents who answered, ‘Yes’ to the question “*Have you heard of the medical condition called sepsis?*”

2 n equals the number of respondents who answered, ‘Yes’ to the question ‘*Have you ever actively looked for information about sepsis?*’ which was only displayed to respondents who answered Yes’ to the question “*Have you heard of the medical condition called sepsis?*”

3 n equals the number of respondents who answered, ‘Yes’ to the question “*Did you experience any difficulties getting the information you wanted or needed about sepsis?*” which was displayed only to respondents who answered, ‘Yes’ to the question ‘*Have you ever actively looked for information about sepsis?*’

4 n equals the number of respondents whose responses to the question “*Where did you look for information about sepsis*?” did not include “the Internet”.
